# Traffic speed prediction techniques in urban environments

**DOI:** 10.1016/j.heliyon.2022.e11847

**Published:** 2022-12-01

**Authors:** Ahmad H. Alomari, Taisir S. Khedaywi, Abdel Rahman O. Marian, Asalah A. Jadah

**Affiliations:** a Yarmouk University (YU), Department of Civil Engineering, P.O. Box 566, Irbid 21163, Jordan; b Department of Civil Engineering, Jordan University of Science & Technology (JUST), P.O. Box 3030, Irbid 22110, Jordan

**Keywords:** Environmental science, Computer science, Speed, Multiple Linear Regression, Machine learning, Artificial Neural Network, Support Vector Machine, Random Forest

## Abstract

The present study developed Multiple Linear Regression (MLR) and machine learning (ML) models, including Artificial Neural Network (ANN), Support Vector Machine (SVM), and Random Forest (RF), to predict the mean free-flow speed (FFS) using several geometric, traffic, and pavement condition variables. The traffic features group includes spot speed, speed limit, average speed, 85th percentile speed, traffic and crossing pedestrian volumes, volume of exiting vehicles, percentage of elderly crossing pedestrians (Elderly%), percentage of heavy vehicles (HV%), and traffic calming measures (TCMs). The geometric characteristics include lateral clearance, number of effective lanes, number of access points (including median openings), road grade, effective lane width, and median width. The pavement condition category includes pavement roughness in the International Roughness Index (IRI). A total of 11 urban arterials were used to develop the MLR model and train the ML models. Test data were collected from two randomly selected roads to evaluate the performance of each model, investigate the differences between conventional linear regression and ML approaches, and determine the best prediction models based on the results of the two techniques. Results showed that the proposed ML algorithms outperformed linear regression models. They are believed to be valuable and strong tools to predict the mean FFS that adapts to sudden changes in traffic flow caused by exogenous conditions on urban arterials and can be employed in determining the most influential factors and building reliable prediction models where spot study is not feasible due to time and resource limitations.

## 1 Introduction

Transportation systems are one of the significant components of the infrastructure in cities and one of the essential elements in their inhabitants' daily lives. Urban streets are used by various individuals and vehicles, including people who walk to work or school, bicycles, buses, emergency vehicles such as fire trucks and ambulances, and cars. If urban streets are not adequately designed, all these vehicles may not travel as effectively as they should. In this context, one of the essential key elements in the design of urban streets is traffic speed, which has been a great concern to urban planners and vehicle engineers. Furthermore, traffic speed affects safety, time, comfort, convenience, and economy (Medina and Tarko, 2005). So, if we could predict the traffic speed effectively, this would benefit drivers and traffic agencies because it would help traffic managers be proactive and implement the best traffic management strategy. Accordingly, accurately forecasting vehicular speeds helps evaluate roadway planning, design, traffic operations, and safety.

High, unsafe speeds occur primarily under free-flow conditions when low-density traffic streams mainly consist of isolated vehicles (Bassani et al., 2014). As a result, free-flow speed (FFS) is an essential element to consider in the traffic research field and practice. FFS and its variability among drivers are believed to be critical safety factors (Medina and Tarko, 2005). The FFS has several definitions depending on the application or context. It is defined as the desirable average speed adopted by the driver when not restricted by other vehicles in the stream under a particular set of road conditions (HCM, 2010). In our circumstance, two key definitions are provided. First, the FFS on an urban roadway is the speed at which a vehicle travels in low-traffic conditions when all of the street's signals are green for the entire trip. The average driver's chosen speed is the FFS when vehicle interaction and traffic control are not involved. Secondly, it is the average speed of the traffic stream when traffic volumes are low enough that drivers are unaffected by the presence of other vehicles and feel comfortable traveling, and when intersection traffic control is absent or far enough away to have little effect on speed choice (HCM, 2010). In theory, FFS should be the speed at which the flow and density are both zero. Observing zero flow and density is, of course, impossible. So, to get around this problem, it is usually monitored at low flow rates. In addition, FFS is critical for traffic stream analysis for incidents and bottlenecks. As a result, the elements that influence its value should be carefully analyzed to comprehend traffic behavior. Traffic stream analysis is also valuable for sensitivity analysis (Rao and Rao, 2015).

Current design policy officials define operating speed as the average speed at which drivers are recorded driving during free-flow conditions (AASHTO, 2001). The 85^th^ percentile speed, a statistical measure representing the speed at or below which 85% of total vehicles are noted traveling past a monitored point under free-flowing conditions, is also used to describe operating speed when the influence of the road environment on speed selection is being assessed.

The maximum or minimum speed in some situations at which vehicles may legally travel on a given road length is called the posted speed. Traffic engineers use the 85th percentile speed to establish the speed limit at a safe level, reducing crashes and ensuring consistent traffic flow along a roadway. According to the Manual on Uniform Traffic Control Devices (MUTCD), agencies should set speed limits within 8 km/h (5 mph) of the 85th percentile speed of traffic under free-flow conditions (MUTCD, 2009). Nevertheless, there are four general approaches to setting speed limits: engineering approach, expert system approach, optimization, and injury minimization or safe system approach (Forbes et al., 2012).

There is limited research on Jordan's urban speeds and road characteristics due to the lack of urban road data. The most recent study in this direction was published by Alomari et al. (2021a; 2021b). Their proposed linear model has a prediction ability restricted to the rural arterials on which the data collected cannot detect any changes in traffic conditions. However, the accuracy of forecasting and prediction has significantly improved because artificial intelligence models such as machine learning (ML) and deep learning improve prediction performance continuously. ML modeling approaches are effective data-mining techniques when investigating traffic speed factors. They can be employed in determining the most influential factors and building reliable prediction models able to detect any change in traffic conditions and give a better understanding of the dynamic behavior of traffic that could improve road safety. Therefore, this research uses ML techniques, including Artificial Neural Network (ANN), Support Vector Machine (SVM), and Random Forest (RF) to look into the speed data obtained across multiple urban arterials in Irbid, Jordan, to achieve the best relationship for safe geometric road design in the future. More specifically, the main objectives of this study can be summarized as follows:

1. Investigate different factors that may cause speed variations and affect the mean FFS on urban streets.
2. Develop statistical models, including traditional linear regression and ML models, representing the relationships between mean FFS and influencing factors.
3. Compare different prediction techniques for predicting traffic speed, based on data gathered from multiple arterials in Irbid city, using typical data collection methods such as traffic radar and manual counting and innovative traffic collection methods such as smartphone applications.
4. Highlight the significance of the ML approach as it is expected that the developed models would be helpful for future prediction of mean FFS when roadway characteristics or traffic operational factors change and where spot study is not feasible due to time and resource limitations.

## 2 Literature review

Many speed prediction techniques were developed, including conventional statistical and artificial intelligence models. However, the traditional statistical models are sometimes unsatisfactory due to the difficulties of accurately reflecting stochastic traffic flow characteristics. The speed of motor vehicles, as mentioned earlier, may depend on the speed limit, operating speed, road design characteristics, traffic volume, weather conditions, vehicle characteristics, or even driver behavior (Leong et al., 2020; Silvano et al., 2020).

Several studies are summarized to understand how traditional statistical models investigated traffic speed factors. Fitzpatrick et al. (2001) used a regression technique to investigate the effects of traffic control devices, geometrics, and roadside characteristics on the operating speed of straight and horizontal curve sections on major suburban four-lane arterials in several areas of Texas (United States). Results showed that the speed limit, access density, and deflection angle were significant variables that explained 75% of the variance of operating speed for the horizontal curves model (R^2^ = 0.75). For straight sections, the posted speed was only included in the model with R^2^ = 0.54. Furthermore, another important finding was how much the posted speed limit was significant in assessing operating speed in straight and horizontal curves and how the R^2^ value improved compared to the models without the posted speed variable.

In Pennsylvania and North Carolina (United States), Himes and Donnell (2010) developed mean operating speed and speed deviation models along rural and urban four-lane highways by considering geometric design features and traffic flow parameters. The importance of this research was in developing four models that could effectively predict the mean speeds and speed deviations of left and right lanes. Moreover, it was evident that the different geometric design characteristics were correlated with mean speed and speed deviation in both the left-lane and right-lane models. Another study in Virginia (United States) by Ali et al. (2007) investigated the interaction between the mean FFS as a dependent variable with the posted speed limit, 85^th^ percentile FFS, and roadway geometric variables as independent variables on 35 four-lane urban roadways. Linear regression models were established to evaluate the relationship between those variables, with R^2^ values around 0.87. According to the findings, segment length, median width, and posted speed all had a significant effect on FFS on urban routes. The previous variables had a positive sign coefficient, indicating that those variables and the mean FFS had a positive relationship.

By carrying out a series of traffic field surveys in Warangal (India), Gulivindala and Mehar (2018) determined the impact of side friction and traffic volume on the mean speed of vehicle traffic streams. The result demonstrated that as side friction increases, vehicle speed drops at all traffic volume levels. At lower levels of side friction, though, no difference in speed was detected. The capacity value for combined data was computed using Greenshields' model (Greenshields et al., 1935), which showed a 9% reduction in the value regardless of whether side friction was considered or not. Another study in Delhi (India) was conducted by Rao and Rao (2015) to see how roadside friction affects road capacity in the Indian metropolis. According to the developed model (R^2^ = 0.541), the following factors were found to have a significant impact on FFS for urban arterials of a particular vehicle type: total vehicles, number of access points, friction points (pedestrian crossings, bus stops), flyovers, and intersections, and section length. There was also a 45–67% reduction in average vehicle speed on portions with on-street parking and a 49–57% reduction on sections with bus stop spots. Lastly, Kadhim et al. (2020) studied the spatial and temporal variation of travel speed on Palestine Street, classified as a major street in Baghdad (Iraq) and surrounded by various mixed land-uses. After dividing the roads into two sections, they created a statistical model (R^2^ = 0.685) using SPSS to predict travel times and delays, including access points for left-turn movements and mixed land use. One essential finding was that the access point has a negative effect on average travel speed, with reductions of 11% and 7% in regions I and II, respectively.

In recent years, neural networks (NN) have been widely used for short- and long-term traffic predictions. In Texas, United States, Vanajakshi and Rilett (2007) compared the performance of the SVM, ANN, and historical and real-time techniques to investigate the performance of the SVM method in the short-term prediction of travel time. The results showed that SVM and ANN performed better in predicting travel time than historical and real-time methods and that these two methods have an excellent dynamic response. Furthermore, despite the substantial performance similarity between SVM and ANN, the SVM technique provided a slight advantage over ANN since SVM is a suitable option for short-term prediction problems when the amount of data is limited or noisy. In another study in California, United States, conducted by Park et al. (2011), a Network Traffic Modeling-Speed Prediction (NNTM-SP) algorithm based on the NN was presented to predict the speed profile in the future, up to 30 min of travel time. The model was trained using historical traffic data and tested using real data. The suggested algorithm produced good predictive performance on real traffic data, and the predicted speed profile demonstrated that the proposed method could accurately forecast dynamic traffic fluctuations. Moreover, a study in Oklahoma (United States) by Singh et al. (2011) used ANN models as an application for predicting the operating speed of two-lane rural highway sites. The researcher developed four models considering four different input parameters, including physical characteristics of the road, accident data, traffic parameters, and pavement condition. The strength of each training and testing stage was evaluated by calculating the Mean Absolute Relative Error (MARE). These models varied as follows: model 1 with an accuracy of 0.97, which consisted of surface width, shoulder type, shoulder width, average daily traffic, skid number, International Roughness Index (IRI), and posted speed limit, without accident data. While model 2 had the same input parameters as model 1, expect posted at a speed limit with an accuracy of 0.915, which shows model 1 performed better than model 2. While models 3 and 4 were more interested in accident data and showed an overall accuracy of 0.975 and 0.941, respectively, meaning that the inclusion of accident data improved the model's performance marginally.

Zou et al. (2022) studied vehicle acceleration prediction using ML models and driving behavior analysis in the United States. The driving behavior semantics are split up using the Finite Mixture of Hidden Markov Model (MHMM). The vehicle acceleration is predicted using Long Short-Term Memory (LSTM) and Gate Recurrent Unit (GRU). The results of the predictions indicate that the technique proposed in this study can enhance the precision of predictions about how fast a vehicle is going by a lot. The study's findings indicated that the MHMM helps analyze personalized driving behavior and that GRU works better than LSTM. In another study by Zhang et al. (2022), they investigated freeway traffic speed prediction using a novel architecture named Temporal Fusion Transformer (TFT). The TFT can measure both short-term and long-term temporal dependence through a multi-head attention mechanism. The prediction model is trained and tested with speed data from an interstate freeway in Minnesota, the United States. Several classic traffic prediction methods are compared to the TFT, and the results show that the TFT does best at predicting speed when the prediction horizon is longer than 30 min.

Worldwide, Semeida (2013) investigated the operating speeds on multi-lane highways in Egypt in two categories: agricultural and desert roads. Linear regression and Multilayer Perceptron (MLP) ANN were used to capture the best technique for predicting the operating speed. The findings showed that the ANN model (R^2^ = 0.978), trained using 35 samples and six samples for testing, provided more reliable results than the regression model (R^2^ = 0.761), at a 95% confidence level in terms of predicting the operating speed. Moreover, the following factors: pavement width, median width, and presence of side access along the section, respectively, were found to have the most significant effect on the operating speed. In contrast, the posted speed limit was discovered to have a negligible impact on the operating speed. The researcher explained this result by mentioning bad driving behavior in Egypt. In another study in Budapest, Hungary, Csikos et al. (2015) suggested a traffic speed prediction algorithm for urban road traffic networks using a congestion forecasting pattern identification method based on the ANN technique. During a VISSIM simulation, 3500 data points were produced, 2500 data points were used for training, and 1000 for testing the learning algorithm. The proposed algorithm was then trained, tested, and analyzed on a real-world test network using actual traffic data from the links and VISSIM. The findings revealed that pattern recognition performance is influenced by the generation and narrowing of the input data. It is also demonstrated that adequate prediction may be achieved in short time intervals, making it a valuable method for traffic management systems. Furthermore, greater data class accuracy will likely result in better prediction performance.

Long Short-Term Neural Network (LSTM NN) was used in Beijing, China by Ma et al. (2015) to forecast traffic speed based on historical data for periods ranging from one to 4 min. The performance metrics Mean Absolute Percentage Error (MAPE) and Mean Squared Error (MSE) have been used. The dataset considering both speed and volume produced better outcomes than the dataset that solely considers speed values. Also, the results suggest that the LSTM NN can make a more accurate and stable prediction output than different typologies of dynamic NN and other commonly used parametric and nonparametric algorithms. One last study by Bratsas et al. (2020) compared different ML methods for traffic speed prediction, including Support Vector Regression (SVR), RF, Multilayer Perceptron (MLP), with Multiple Linear Regression (MLR) based on different scenarios and a fleet of 1200 taxis. The findings showed that while the SVR model performs better under stable conditions with slight variations, the MLP model responds better to cases with more significant variations and fewer errors.

Summing up the above literature review, ML modeling methodologies are robust data-mining tools when looking into elements that affect traffic speed. They can be used to identify the most relevant factors and create accurate prediction models to detect any changes in traffic conditions. As a result, this study adopts these methodologies to look at traffic data collected from different urban arterials in Irbid, Jordan, to find the optimal relationship for future safely geometric road design. Furthermore, there is limited research on Jordan's urban speeds and road characteristics due to a lack of urban road geometry and speed data. No previous study considered geometric, traffic, and pavement condition parameters and the absolute difference between the 85th percentile speed and the posted speed limit. Previous studies also did not develop ML models to predict the mean FFS based on these factors.

## 3 Methodology

### 3.1 Sections selection criteria

Eleven urban streets (22 streets considering both directions) were selected with different geometric and operational characteristics. The following are the selection criteria set for the study arterials:

1. The study arterial should have good pavement condition with various degrees of roughness but not deteriorated as the deteriorated sections do not represent the actual driving behavior.
2. The study section is almost straight, with no sharp horizontal curves or steep slopes.
3. The study arterial should be homogeneous in both traffic and geometric characteristics. If not homogeneous, then the segmenting takes place.
4. The study section must be long enough to allow vehicles to reach their desired speeds if two intersections delimit it with traffic control devices. There is no minimum length requirement for a study section defined by two intersections without traffic control devices. However, it must be positioned far enough away from nearby traffic control devices to neglect their effects during the study.

The first step in selecting the study sections was scanning study arterials using Google Earth to locate a wide range of roadways with different geometric and traffic characteristics. The second step was followed by site visits to observe any other influencing factors in site selection, such as pavement condition, traffic calming measures (TCMs) such as speed humps and cat-eye reflectors, and the presence of traffic control devices.

### 3.2 Data collection methodology

Vehicle speeds were monitored on sunny days with dry pavement conditions using an LTI-20-20-MARKSMAN laser radar gun (Laser Technology, 2013). A second observer surveyed the number of exiting vehicles, traffic volume, and crosswalk volume. Field measurements were taken during morning off-peak periods from 11 urban arterials to ensure free-flow traffic movement without any traffic congestion or incidents for 2 h in each direction, with 12 intervals per direction, each equal to 10 min in duration, resulting in 264 overall study intervals, then the average speed was calculated in each interval to produce 264 data points that were used as a training data set. Additional field data were observed along with the speeds to cover different aspects such as speed limit, TCMs, traffic and pedestrian volumes, lateral clearance, number of lanes and access points, road grade, effective lane width, median width, and pavement roughness in terms of the International Roughness Index (IRI). These variables, along with the computed 85th percentile speed, percentage of heavy vehicles (HV%), and percentage of elderly crossing pedestrians (Elderly%), were used as inputs (independent variables) to our models to predict traffic speed (dependent variable) accurately. After finishing data collection, the minimum sample size for each study location was verified using Eq. (1) (Garber and Hoel, 2018) and Eq. (2) (Israel, 1992). The data was then filtered, characterized, coded, and statistically analyzed using SPSS and MATLAB statistical software Version R2021.

(1) $$N={\left(\frac{ZS}{d}\right)}^{2}$$

(2) $$N=\left(\frac{N_{p}}{1+N_{p}e^{2}}\right)$$

Where:

− N = Minimum sample size.
− N_P_ = Population size.
− Z = Number of standard deviations corresponding to the required confidence (±1.96 for 95% confidence level) or is the critical values of the normal curve that cuts off an area α at the tails.
− S = Sample standard deviation (km/h).
− d = limit of acceptable error in the average speed (km/h).
− e = is the desired level of precision (±5% is commonly used).

### 3.3 Pavement roughness and road grade data collection

Using smartphones to collect data is a promising alternative because of their low cost and easy-to-use features, in addition to their potentially comprehensive population coverage as probe devices. Formulating FFS model based on local traffic conditions that can properly predict FFS without the need for field measurements is crucial for saving data collection time and expenses (Leong et al., 2020). Technologically speaking, smartphones now include accelerometers and Global Positioning System (GPS) sensors. Without expensive methods, these sensors could gather information about variables that may influence the FFS, such as pavement roughness data and road grade (slope). Consequently, a timesaving, low-cost, and precise technique is crucial in a developing country like Jordan.

Several studies used smartphone applications to measure pavement roughness and found promising results (Douangphachanh and Oneyama, 2014; Islam et al., 2014; Alatoom and Obaidat, 2021; ALQaydi et al., 2021; Ekpenyong et al., 2021; Zhang et al., 2021) with the assumption that a rough evaluation of road surface quality via a smartphone would be helpful for transportation managers and planners. Nevertheless, calibration is needed to improve the accuracy of the collected roughness data.

**Figure 1:**
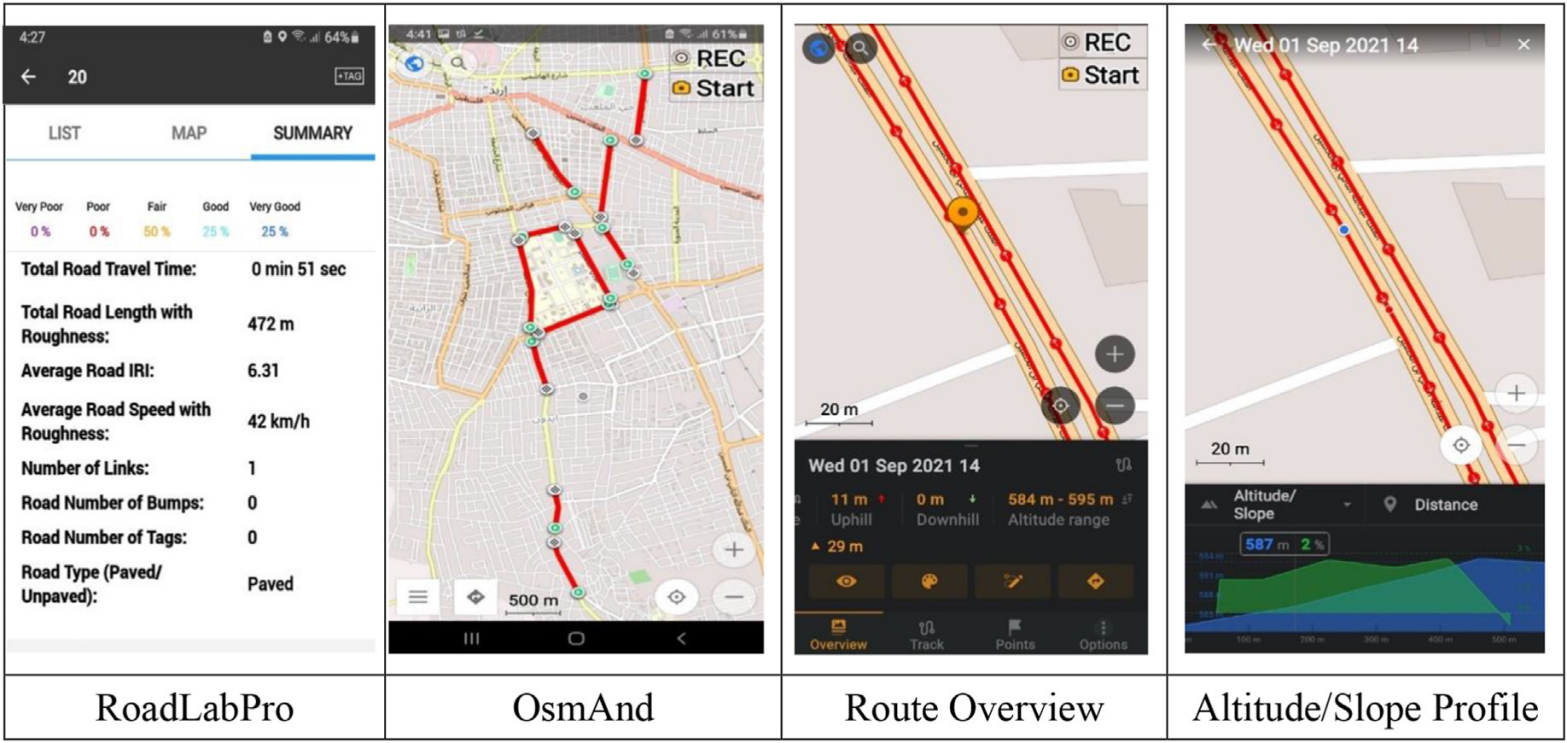
RoadLabPro and OsmAnd.

One of the standard apps is RoadLabPro (Figure 1), a free smartphone-based road condition mapping and monitoring application developed by the World Bank (World Bank, 2018). The application algorithm uses data from the phone's accelerometer, GPS, and gyroscope sensors to evaluate pavement roughness automatically.

To evaluate pavement roughness, a smartphone was mounted securely and in a well-oriented position on a vent mount at the front of the testing vehicle traveling at the posted speed limit of the study section. The reason for choosing this position was based on the findings of a study conducted in Jordan's urban streets (Alatoom and Obaidat, 2021), which revealed that IRI estimated from vent mount produced better results than IRI estimated utilizing free condition. The operational speed for RoadLabPro is within 15–100 km/h, and since the study sections have posted speed limits ranging from 40-60 km/h, driving the test vehicle within this range should not be a problem.

OsmAnd, on the other hand, is a map application that provides free, high-quality data from around the world (OsmAnd, 2010). The "'Plan route" feature in OsmAnd is a powerful tool that allows users to create GPX tracks or add new segments, measure distances on the map, snap track to the nearest available road with one of the navigation profiles, and view the route's elevation and slope profile in an interactive manner allowing to see the data for a specific point on the route. An example of an elevation and slope profile obtained from a study section is shown in Figure 1.

### 3.4 Descriptive statistics of the main variables

A descriptive statistical analysis was performed to describe the statistical characteristics of the main variables included in this study. The collected data was classified and summarized to show the concentration and dispersion of each parameter. Descriptive statistics for all the models’ parameters are given in Table 1.

**Table 1:** Descriptive statistics.

Parameter	N	Mean	Std. Dev	Variance	Range	Min	Max
Traffic Mean FFS (Km/h)	264	41.76	4.95	24.54	23.32	32.14	55.46
Passenger Car (PC) Mean FFS (Km/h)		42.58	5.00	24.98	24.16	32.88	57.04
HV Mean FFS (Km/h)		36.98	5.20	27.00	28.83	25.67	54.50
Section Length (m)		443.18	111.18	12361.70	350.00	250	600
|V85-PS| PC (Km/h)		9.12	5.33	28.46	22.00	0.00	22.00
|V85-PS| HV (Km/h)		4.73	2.98	8.88	12.40	0.00	12.40
Tot Vol (Veh/10min)		49.03	6.59	43.40	30.00	36	66
HV (%)		16.45	5.09	25.87	25.93	4.26	30.19
Vehicle Turning and Exit from Section (Veh)		5.72	4.84	23.40	18.00	0	18
Crossing Ped Vol (Ped/10min)		9.66	5.62	31.61	20.00	0	20
Elderly (%)		30.31	26.18	685.37	100.00	0.00	100.00
Number of Lanes		1.84	0.47	0.23	2.00	1	3
Effective Lane Width (m)		4.52	0.89	0.79	2.54	3.16	5.70
Median Width (m)		1.76	0.85	0.72	3.30	0.50	3.80
Lateral Clearance (m)		4.84	0.97	0.95	3.16	3.16	6.32
Density of Access Points (#/Km)		5.25	4.18	17.43	16.00	0.00	16.00
Avg IRI (m/Km)		6.38	0.90	0.81	4.82	3.31	8.13
Slope (%)		0.02	2.44	5.96	10.28	-4.85	5.43
Traffic Calming Measures (TCMs: L, M, H)		-	-	-	-	-	-

It is essential to mention that the absolute difference value between the 85^th^ percentile speed and the posted speed limit |V85-PS| was introduced as one variable because the range and variation of the speed limit are limited for the selected arterials, ranging from 40 km/h to 60 km/h, and since it is believed that the posted speed limit has a direct influence on the desired speed or has a high correlation on the operating speed (Medina and Tarko, 2005; Ali et al., 2007; Singh et al., 2011). Consequently, the posted speed limit could be entered into the models without separating models (i.e., models with PS and models without PS). Moreover, the absolute difference between the speed limit and the 85th percentile speed/design speed has been discovered to substantially affect the case of killed or seriously injured accidents (Hashim, 2006). Also, Alomari et al. (2021a; 2021b) concluded that the difference between design speed and the speed limit has a considerable effect on the speed variance. As a result, the absolute difference value of "V85-PS" is a crucial factor to think about.

Effective lane width and the number of effective lanes were proposed in the study to consider the effect of the on-street stopped vehicles, which served as side friction that often conflicts with the traffic flow and influences the selected speed (Rao and Rao, 2015). Therefore, the width of those vehicles was subtracted directly from the actual lane width in the field by a measuring tape. Median openings on the opposite side that are expected to affect traffic flow in the direction of interest significantly were combined with access point counts.

## 4 Analysis and results

**Figure 2:**
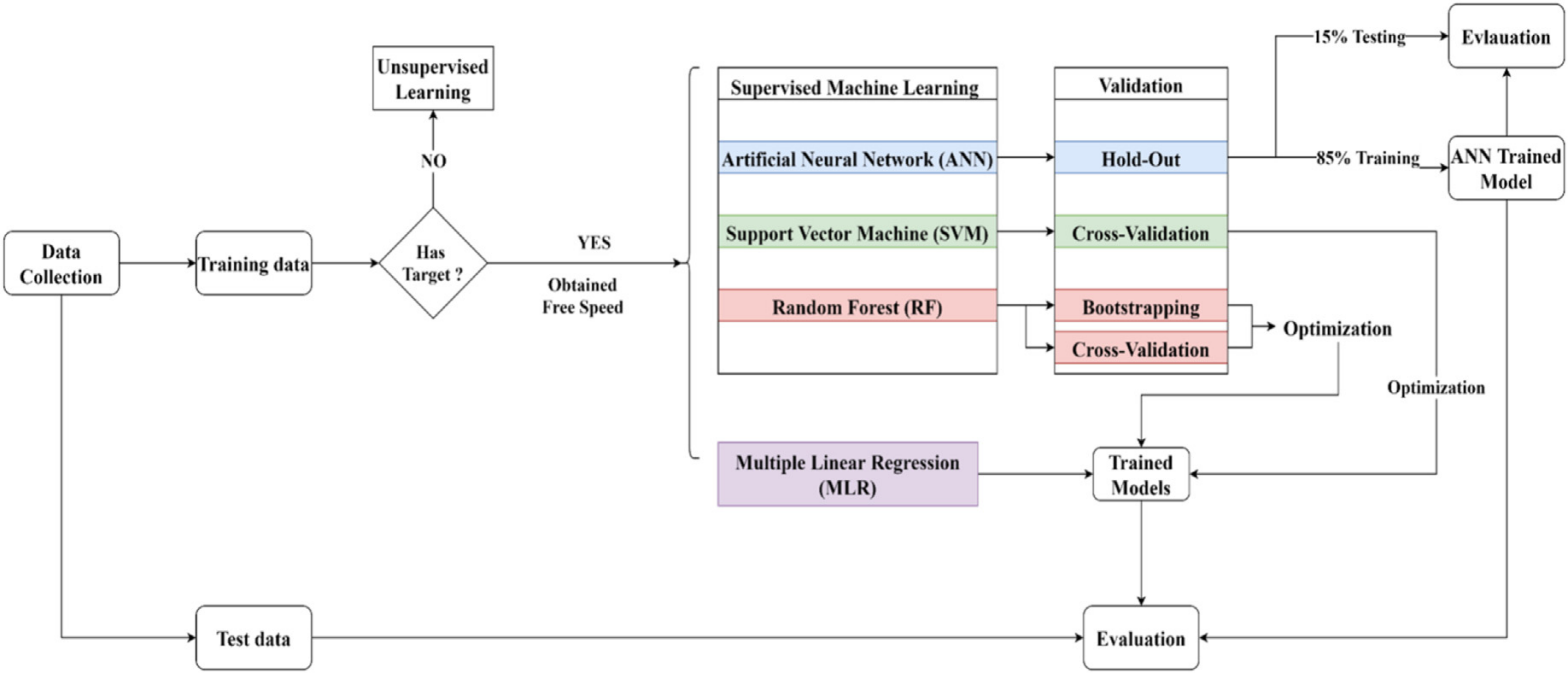
Analysis flow chart.

Two different techniques were proposed in this research to develop the prediction models. The first technique is the multiple linear regression method. The second one is ML techniques, including ANNs, SVMs, and RF. Figure 2 below summarizes the methodology used in the model development process in this study.

The coefficient of correlation between the dependent and independent variables is required to develop any regression model. This information is critical in determining which independent variables should be included in the regression model. It is also essential to understand the intercorrelation between the independent variables. The correlation matrix between the independent variables in this study is shown in Table 2. From the correlation matrix, the highest intercorrelation between the independent variables was found between the Number of Lanes and Eff Lane width, with a correlation coefficient equal to 0.68. The reason is that when effective lane width increases, the number of lanes increases, at least in Jordan. Thus, they are strongly and positively correlated with each other. The second highest intercorrelation was between median width and effect of TCMs, with a correlation coefficient equal to -0.63. The negative relation can be interpreted as when the effect of TCMs declines, the speed will increase, and the gap decreases in the main traffic stream. Hence, the pedestrians need a higher median width to wait and find a suitable gap to cross the road. Otherwise, it can be said there is no or weak correlation between the independent variables.

**Table 2:** Correlation matrix.

	Sec. Leng	|V85-PS| PC	|V85-PS| HV	Tot. Vol	HV%	Exit. Vol	Cross Ped Vol	Eld %	# of Lanes	Eff. Lane width	Med. width	Lat. Clear	Density of APs	Avg IRI	Slope%	Effect of TCMs
Section Length	1	-0.14	0.02	-0.12	0.06	-0.25	-0.17	0.02	-0.04	0.28	-0.13	-0.03	-0.37	0.18	0.03	-0.18
|V85-PS| PC	-0.14	1	-0.24	0.2	0.07	0.09	0.17	0	-0.02	0.09	0.19	-0.07	0.11	-0.09	-0.08	-0.12
|V85-PS| HV	0.02	-0.24	1	-0.08	-0.1	-0.1	-0.14	0.09	-0.07	0.08	-0.05	0.18	-0.15	0.02	-0.08	0.21
Tot Vol	-0.12	0.2	-0.08	1	0.05	0.02	0.11	0.08	0.15	0.11	0.17	-0.01	0.15	-0.06	-0.01	-0.18
HV%	0.06	0.07	-0.1	0.05	1	-0.09	0.04	0.04	0.01	0.08	0.06	0.01	0.05	0.04	0.21	-0.16
Exiting Vol	-0.25	0.09	-0.1	0.02	-0.09	1	0.03	0.01	-0.1	-0.05	-0.04	-0.22	0.43	-0.22	0.06	-0.03
Crossing Ped Vol	-0.17	0.17	-0.14	0.11	0.04	0.03	1	-0.12	0.14	0.1	0.11	0.15	0	-0.06	-0.14	-0.02
Elderly%	0.02	0	0.09	0.08	0.04	0.01	-0.12	1	0.01	-0.02	-0.01	-0.04	-0.05	0.01	0.04	-0.03
# of Lanes	-0.04	-0.02	-0.07	0.15	0.01	-0.1	0.14	0.01	1	**0.68**	0.32	0.33	-0.23	0.42	-0.11	-0.02
Eff Lane width	0.28	0.09	0.08	0.11	0.08	-0.05	0.1	-0.02	0.68	1	0.18	0.38	-0.24	0.16	-0.09	-0.12
Med. width	-0.13	0.19	-0.05	0.17	0.06	-0.04	0.11	-0.01	0.32	0.18	1	0.47	-0.17	0.03	0.01	-0.63
Lateral Clear.	-0.03	-0.07	0.18	-0.01	0.01	-0.22	0.15	-0.04	0.33	0.38	0.47	1	-0.51	0.11	-0.07	-0.14
Density of APs	-0.37	0.11	-0.15	0.15	0.05	0.43	0	-0.05	-0.23	-0.24	-0.17	-0.51	1	-0.24	-0.05	-0.01
Avg IRI	0.18	-0.09	0.02	-0.06	0.04	-0.22	-0.06	0.01	0.42	0.16	0.03	0.11	-0.24	1	0.11	0.39
Slope%	0.03	-0.08	-0.08	-0.01	0.21	0.06	-0.14	0.04	-0.11	-0.09	0.01	-0.07	-0.05	0.11	1	-0.09
Effect of TCMs	-0.18	-0.12	0.21	-0.18	-0.16	-0.03	-0.02	-0.03	-0.02	-0.12	**-0.63**	-0.14	-0.01	0.39	-0.09	1

### 4.1 Multiple Linear Regression (MLR)

Using SPSS software (IBM, 2009), the regression analysis approach was divided into three parts (see Table 1):

– Traffic mean FFS is DV, and other factors are IVs.
– PC mean FFS is DV, and other factors are IVs except |V85-PS| HV.
– HV mean FFS is DV, and other factors are IVs except |V85-PS| PC.

Since some input variables do not have an essential explanatory effect on the target variable, the stepwise regression method was adopted. It keeps only the statistically significant terms in the model instead of using all independent variables, and the variables with high collinearity will be automatically excluded from the model. Many speed models were developed for Traffic, PC, and HV. However, the minimum Mean Square Error (MSE) and highest R^2^ value model is selected. The form of multiple linear regression models is shown Eq. (3), and the MSE defined as shown in Eq. (4).

(3) $$Y=\beta_{o}+\sum \beta_{i}X_{i}$$

(4) $$MSE=\frac{1}{n}\sum_{i=1}^{n}{\left(y_{\left(act\right)i}-y_{\left(pred\right)i}\right)}^{2}$$

Where:

− Y: Target variable.
− X_i_: Independent variables.
− β_o_: Regression constant.
− β_i_: Regression coefficients.
− y_(act)_: is the actual response.
− y_(pred)_: is the predicted response.

Tables 3 and 4 show the model's summary, Analysis of Variance (ANOVA), the test of normality, and model's coefficient. Based on model's coefficients, the following models were obtained:

(5) $$FFS_{Traffic}=43.684-0.666{\left|V85-PS\right|}_{PC}+1.706\left(Eff\;LW\right)+2.025\left(L\right)-1.412\left(IRI\right)+0.269\left(DAPs\right)+0.777\left(Lat\;Clear\right)-0.084\left(Vol\right)+1.882\left(\#Lanes\right)+0.184\left(slope\right)$$

(6) $$FFS_{PC}=44.618-0.737{\left|V85-PS\right|}_{PC}+1.875\left(Eff\;LW\right)-1.525\left(IRI\right)+0.254\left(DAPs\right)+1.290\left(Lat\;Clear\right)-3.085\left(M\right)+0.249\left(slope\right)+0.103\left(Exiting\;Vol\right)-0.066\left(Vol\right)+1.237\left(\#Lanes\right)$$

(7) $$FFS_{HV}=39.037-9.835\left(H\right)+0.4{\left|V85-PS\right|}_{HV}+4.281\left(\#Lanes\right)-2.106\left(MW\right)-0.154\left(Vol\right)+0.141\left(DAPs\right)$$

Where:

− FFS: Mean Free-Flow Speed.
− Eff LW: Effective Lane Width.
− No. of Lanes: Number of effective lanes.
− MW: Median Width.
− L, M, H: Effect of TCMs as: Low, Medium, High, respectively. This variable was introduced as a dummy variable.
− DAPs: Density of Access Points.
− Lat Clear: Total Lateral Clearance (left lateral clearance plus right lateral clearance).
− Vol: Total traffic volume in 10-min interval.
− Exiting Vol: Total exiting traffic volume in 10-min interval.
− Refer to Table 1 for the descriptive statistics and the measuring units for each variable.

**Table 3:** Model summary, ANOVA table, and normality of unstandardized residuals.

Model Summary						
Speed Models	R	R^2^	Adjusted R^2^	Std. Error		Durbin-Watson
Overall Traffic	0.841	0.707	0.696	2.73029		0.677
PC	0.844	0.713	0.702	2.73077		0.739
HV	0.639	0.408	0.394	4.04525		1.089

**ANOVA Table**						

**Speed Models**	**Sum of Squares**		**df**	**Mean Square**	**F**	**p-value (Sig.)**

Overall Traffic	Regression	4561.306	9	506.812	67.988	.000
	Residual	1893.437	254	7.454		
	Total	6454.744	263			
PC	Regression	4683.863	10	468.386	62.811	.000
	Residual	1886.652	253	7.457		
	Total	6570.515	263			
HV	Regression	2895.240	6	482.540	29.488	.000
	Residual	4205.559	257	16.364		
	Total	7100.799	263			
**Normality of Unstandardized Residuals**						

	**Kolmogorov-Smirnov**			**Shapiro-Wilk**		

Speed Models	Statistic	df	p-value (Sig.)	Statistic	df	p-value (Sig.)
Overall Traffic	0.040	264	.200	0.991	264	0.125
PC	0.030	264	.200	0.995	264	0.527
HV	0.038	264	.200	0.993	264	0.247

**Table 4:** Models coefficients.

Speed Models	Entered Variables	Unstandardized Coefficients		Standardized Coefficients	t	p-value (Sig.)	Collinearity Statistics	
		B	Std. Error	Beta			Tolerance	VIF
Overall Traffic	(Constant)	43.684	2.262		19.315	0.000		
	|V85-PS| PC	-0.666	0.035	-0.718	-19.246	0.000	0.831	1.204
	Eff Lane width	1.706	0.276	0.306	6.181	0.000	0.472	2.120
	L	2.025	0.401	0.204	5.044	0.000	0.707	1.415
	Avg IRI	-1.412	0.240	-0.256	-5.878	0.000	0.609	1.641
	Density of APs	0.269	0.049	0.227	5.512	0.000	0.682	1.466
	Lateral Clearance	0.777	0.214	0.153	3.631	0.000	0.652	1.533
	Vol	-0.084	0.027	-0.111	-3.086	0.002	0.886	1.128
	# of Lanes	1.882	0.576	0.180	3.266	0.001	0.378	2.644
	slope (%)	0.184	0.072	0.091	2.569	0.011	0.930	1.076
PC	(Constant)	44.618	2.256		19.776	0.000		
	|V85-PS| PC	-0.737	0.039	-0.786	-18.655	0.000	0.639	1.566
	Eff Lane width	1.875	0.282	0.333	6.661	0.000	0.453	2.205
	Avg IRI	-1.525	0.232	-0.274	-6.577	0.000	0.654	1.529
	Density of APs	0.254	0.053	0.212	4.825	0.000	0.588	1.701
	Lateral Clearance	1.290	0.231	0.251	5.574	0.000	0.558	1.791
	M	-3.085	0.487	-0.304	-6.340	0.000	0.494	2.026
	slope (%)	0.249	0.072	0.122	3.481	0.001	0.927	1.079
	Exiting Vol	0.103	0.041	0.100	2.502	0.013	0.716	1.397
	Vol	-0.066	0.027	-0.087	-2.439	0.015	0.890	1.123
	# of Lanes	1.237	0.624	0.118	1.981	0.049	0.322	3.102
HV	(Constant)	39.037	2.113		18.473	0.000		
	H	-9.835	0.858	-0.651	-11.469	0.000	0.716	1.397
	|V85-PS| HV	0.400	0.085	0.230	4.695	0.000	0.963	1.039
	# of Lanes	4.281	0.608	0.391	7.045	0.000	0.747	1.339
	Median width	-2.106	0.365	-0.343	-5.764	0.000	0.651	1.535
	Vol	-0.154	0.040	-0.195	-3.882	0.000	0.917	1.090
	Density of APs	0.141	0.064	0.113	2.188	0.030	0.863	1.158

The regression models, intercept coefficient (constant), and variables coefficients were significant (P-value less than 0.05) at 95% confidence level as seen in Tables 3 and 4. The value of R^2^ were: 0.707, 0.713, 0.408 for traffic, PC, and HV mean FFS models, respectively. The low value of R^2^ of 0.408 is due to the lack of HV data points compared to PC, as illustrated in the measured sample size in Table 3. Several precautions are taken into consideration to ensure the integrity of the models as follows:

1. **Models’ Logic**

The signs of the multiple linear regression coefficients should agree with intuitive engineering judgment. From the MLR models, it should be noted that the mean FFS increases when DAPs increase. The reason is that when the number of access points increases per unit length of the street, the number of observed exiting vehicles is greater than the number of entering vehicles, which yields more space and free movement in the main traffic stream, especially in the off-peak periods.

There was a negative correlation between |V85-PS| and the mean FFS. Because when the mean FFS and V85 increase, which most likely happens in off-peak periods, the V85 approaches PS; as a result, the absolute difference decreases except for Eq. (7). This can be explained by the aggressive behavior of Jordanian drivers in general and HV drivers in specific (especially the bus drivers), who were noticed to drive above the speed limit; therefore, the absolute difference increases. Generally speaking, Jordanian drivers were aggressive when driving (Abojaradeh et al., 2014; Naghawi and Bannoura, 2019; Atieh et al., 2020; Bener et al., 2020; Al-Mestarehi et al., 2021; Magableh et al., 2017). According to a study by Jadaan et al. (2021), speeding is the most common traffic violation in Jordan; based on the study findings, 30% of drivers tend to violate speed limits early in the morning and late at night. They also have a habit of violating speed limits on major routes.

When slope increases, the mean FFS increases with coefficients of 0.184 and 0.249 for Eqs. (5) and (6), respectively. This result can also be related to Jordanian drivers’ behavior. They were observed to accelerate on the upgrade sections to maintain a relatively high speed to climb and vice versa. Upgrade sections need the engine to operate additional work against gravity in the direction of vehicle motion, which is one crucial variable that impacts engine performance. Nonetheless, the relation between on-road behavioral patterns and real-world road grade, as well as the subsequent impact on energy use and emissions, have received little research (Liu, 2018). The lane width, number of lanes, exiting Volumes, and Low TCMs positively correlate with the mean FFS. While IRI, Vol, MW, and Medium and High TCMs have a negative relation with the mean FFS. All of which are consistent with logic.

2. **Multicollinearity Check**

There should be no multicollinearity among the final selected independent variables (Daoud, 2017). For predictors not to be inter-correlated: Variance Inflation Factor (VIF) should be <10 or Tolerance (reciprocal of VIF) should be >0.1 (O'brien, 2007; Thompson et al., 2017). As seen from Table 4, the specifications are applicable in all cases.

3. **Normality of Residuals**

The Shapiro-Wilk and Kolmogorov-Smirnov tests were used to test the normality of the residuals (Razali et al., 2011). From Table 3, it can be concluded that the residuals are normally distributed since P-value >0.05, which means accepting the null hypothesis that assumes the residuals are normally distributed. Although the Kolmogorov-Smirnov test is recommended for a larger dataset, it was also applicable. Moreover, the frequency histograms for the developed models were plotted in Appendix, Figures A.1 to A.3 to check the normality of the model residuals.

4. **Autocorrelation of Residuals**

The Durbin-Watson statistic is a number that tests for autocorrelation in the residuals from statistical regression analysis (Ali, 1987). Since values in Table 3 are less than 2, indicating positive autocorrelation in the residuals. However, testing for autocorrelation is a commonly used task for researchers dealing with time-series data.

### 4.2 Machine Learning Models

#### 4.2.1 Artificial Neural Network (ANN)

The Multilayer Perceptron (MLP), which comprises one or more hidden layers (Haykin, 1999), is a feedforward ANN model that can deal with non-linearity and is commonly used in engineering applications since it allows for the use of a variety of learning algorithms (Semeida, 2013). The weights of all the neurons in an ANN are typically randomized at the start. This means they have not learned anything yet and must be taught (trained) how to address the problem for which they were created. The inputs are received by the input layer, which is then carried forward through the MLP by taking the dot product of each layer with the weights of the following layer. This dot product yields some values, which are subsequently processed by an activation function. The technique is continued for the subsequent layers until the output layer is reached. The output is compared to the desired (target) value during the model's training process, and an error is calculated using the loss function. This error is then propagated back through the network using a training algorithm, the Bayesian Regularization (TRAINBR) training algorithm in this study, to adjust the connection weights on all links starting at the output layer and heading down to the input layer. This procedure of forwarding and backward correction (updating) of link weights is repeated until a satisfactory level of performance is reached. The training is then completed, and the network will begin making predictions using the final weights (Gardner and Dorling, 1998; Basheer and Hajmeer, 2000).

In this research, MLP ANNs models with a Tansigmoidal (TANSIG) activation function, Eq. (8), gave the best performance of all models based on trial and error approach among other activation functions, which is a non-linear differentiable function that can be used widely in regression analysis (non-classification problems) to ensure effective learning, (Arslankaya, 2020; Alatoom and Al-Suleiman (Obaidat), 2021), and can be defined as shown in Eq. (8). TANSIG(x) is a hyperbolic tangent function which also represents the output of the neuron in the range −1 to 1, x is the input value of that neuron which represents the dot product of each input value (x_i_) from previous neurons in the previous layer with the corresponding weight (w_i_) between the neuron and the previous neurons in the previous layer plus constant or bias (b) as shown in Eq. (9).

(8) $$y=TANSIG\left(x\right)=\frac{2}{1+e^{-2x}}-1$$

(9) $$x=b+\sum_{i=1}^{n}X_{i}W_{i}$$

**Figure 3:**
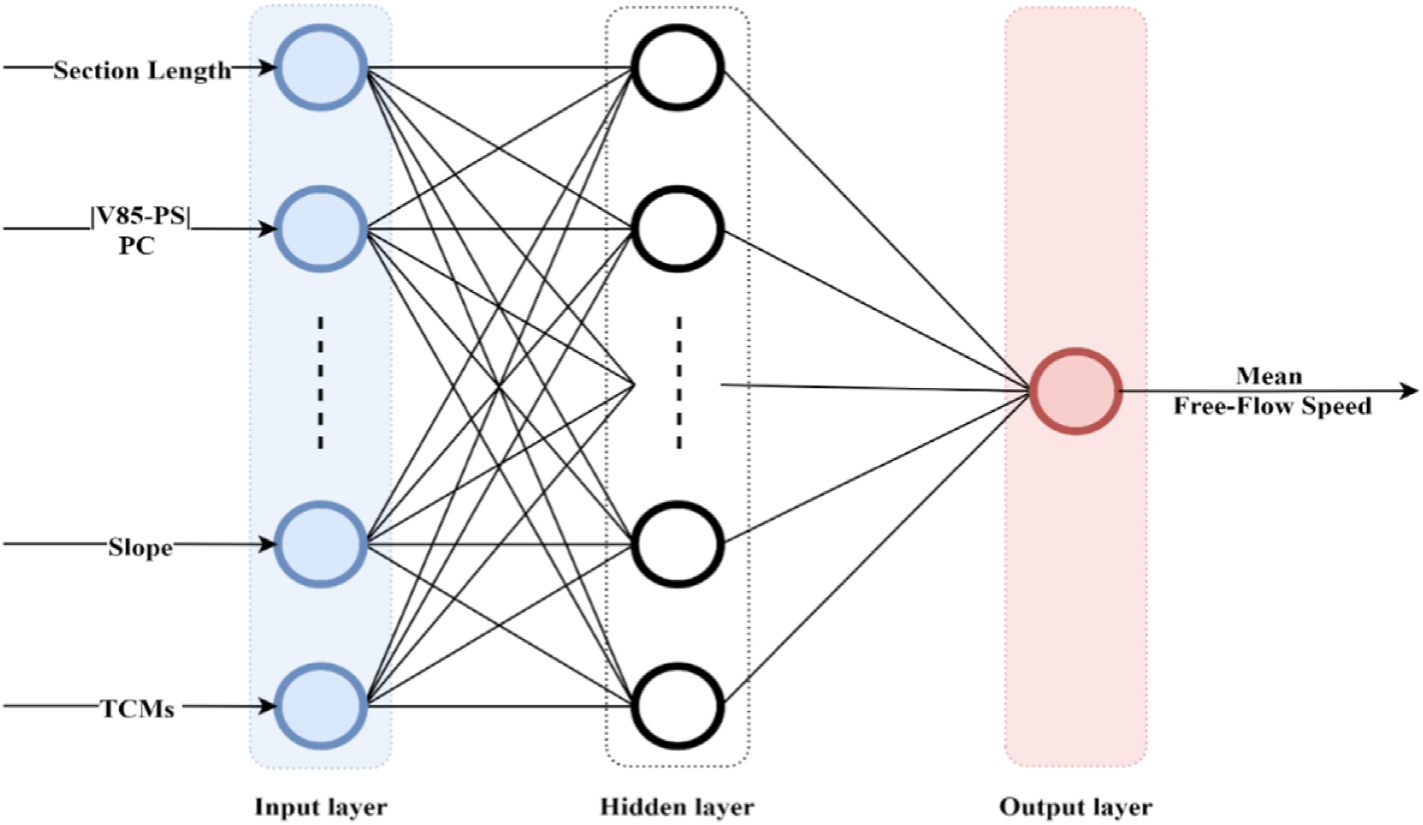
Model development architecture.

In Appendix, Figure A.4 shows the mechanism of the activation function. Non-linear activation functions are required in ANN since most real-world situations are extremely complex. Without non-linear activation functions, a neural network is merely a straightforward linear regression model. This is because linear activation functions can be coupled to create another single linear function. Thus, the entire network will essentially be reduced to a single neuron with the combined linear function as its activation function. That single neuron will not be able to understand complex relationships in data. For most applications, it is believed that a single-layer NN with a sufficient number of hidden neurons will produce a good approximation (Hornik et al., 1989). The architecture of the MLP ANN models is shown in Figure 3.

During the initial model development phase, the entire set of observations is divided into training and testing data sets. The data set contains observations collected from the 11 arterials, while the testing data set contains observations collected from another two randomly selected arterials that will be used later to make the final evaluation and choose the best model. During ANN model development, the training data was further divided into training and testing sets (hold-out set) as part of hold-out validation. The separation was done randomly and in a manner such that the training dataset has the range of variables seen in the testing dataset or expected to be seen in further applications of the model. As a result, numerous trials were carried out to achieve this percentage between training and testing data. Because many earlier studies primarily employed ratios of 70/30, 80/20, and 90/10 (training/testing) (Nguyen et al., 2021), the 85:15 splitting ratio was appropriate and provided the best model performance in this study.

Furthermore, this technique is carried out to avoid the network from the overfitting phenomenon, which occurs when the machine attempts to memorize the training data by utilizing an excessive and unneeded number of training cycles or by attempting large numbers of hidden nodes. Therefore, to achieve the most outstanding performance for both training and testing data, the overall observations were randomized before training the network, along with the Bayesian Regularization (TRAINBR) algorithm, which has been demonstrated to help reduce overfitting (Cawley and Talbot, 2007).

In this research, the appropriate MLP models were built and trained using the MATLAB "nntool" package. In order to achieve the optimal model performance, many trials and iterations were performed, such as adjusting the number of the hidden layer(s) and neurons within the hidden layer, changing the activation function, varying the initial weights, and so on. The performance of the best models for training and testing datasets is provided in Table 5 as a consequence of training and testing processing. The MSE measure, defined earlier in Eq. (4), was utilized to evaluate the performance.

**Table 5:** Performance summary of MLP ANNs.

No. of neurons in hidden layer (N)	Mean FFS Models	Traffic		PC	HV
	ANN Architecture (Input-Hidden-Output)	18-N-1		17-N-1	17-N-1
9	MSE	0.64918		0.93126	4.024
	R	Training	0.98717	0.98105	0.92031
		Testing	0.91709	0.94705	0.83045
		Overall	0.97808	0.97549	0.90281
10	MSE	0.56966	0.55523	3.6701	
	R	Training	0.98846	0.98934	0.93048
		Testing	0.94923	0.89637	0.80832
		Overall	0.98144	0.97825	0.91161
11	MSE	0.68429	0.64816	4.0056	
	R	Training	0.98648	0.98738	0.92584
		Testing	0.95099	0.93523	0.73915
		Overall	0.98058	0.97975	0.89932
Best Model (with10 neurons)	R^2^	Training	0.97705	0.97879	0.86579
		Testing	0.90104	0.80348	0.65338
		Overall	0.96322	0.95697	0.83103

As seen from Table 5, there is no rule for the optimal number of neurons in the hidden layer in general. Even though the results are somewhat consistent with a common practice of using half of the total number of neurons in the input and output layers that most likely would give the best result in terms of MSE and R or R^2^ (Semeida et al., 2010), i.e., at ten neurons with lowest MSE of: 0.56966, 0.55523, 3.6701 and R of: 0.98144, 0.97825, 0.91161 for traffic, PC, and HV respectively. The regression plots at ten neurons are demonstrated further in Appendix, from Figure A.5 through Figure A.7.

#### 4.2.2 Support Vector Regression (SVR)

**Figure 4:**
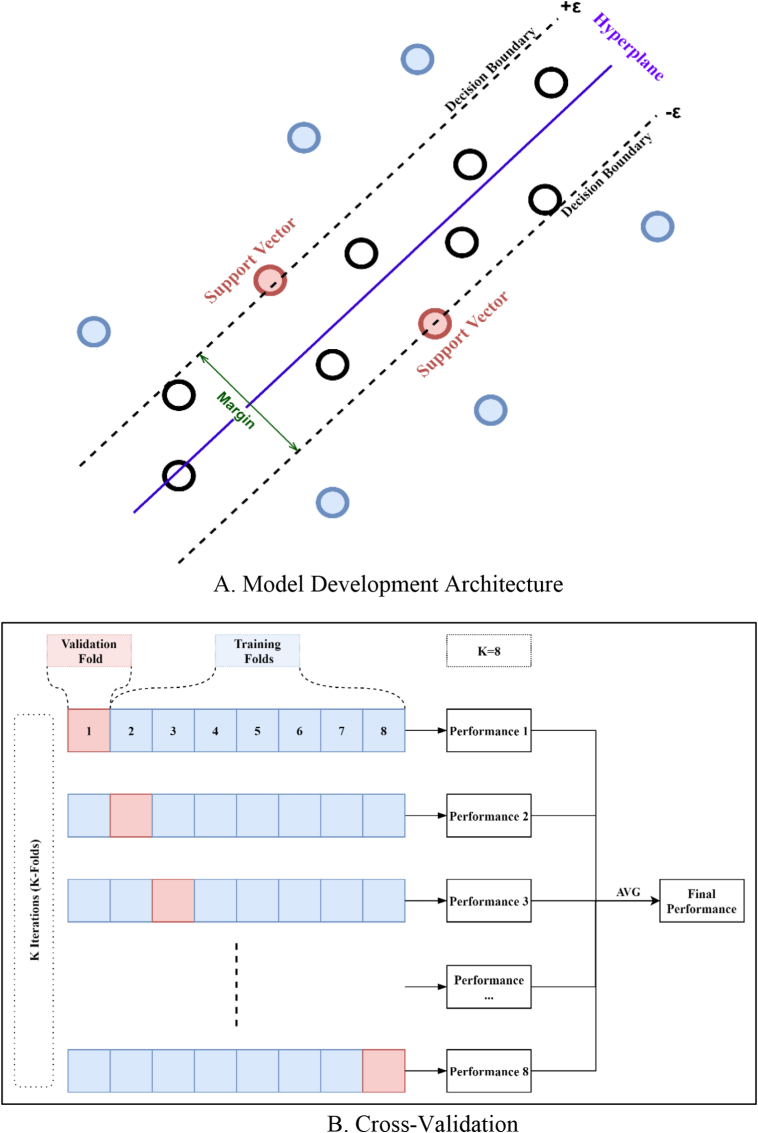
Support vector regression (SVR).

SVMs are supervised ML models that analyze data for classification and regression analysis. Support Vector Regression SVR is the name given to the SVM regression algorithm which is used to predict continuous or discrete variables. SVR's primary goal is to select the best fit line and sometimes uses Kernel transformation functions to produce a roughly linear separation if a non-linear scenario happens. The hyperplane with the greatest number of points in SVR is the best fit line (Awad and Khanna, 2015). The SVR, unlike other regression models, seeks to fit the best line within a threshold value (the distance between the hyperplane and the boundary line "epsilon") rather than minimizing the error between the actual and predicted value. To predict the value, it uses the points with this boundary. Figure 4A shows SVR algorithm (Appendix, Figure A.8). To understand Figure 4A, the following terms should be defined:

1. Hyperplane: In SVM, it is a separation line between the data classes in a higher dimension than the actual dimension. In SVR, it is the line that helps to predict the target value (continuous value).
2. Kernel: The function, such as Polynomial Kernel, Gaussian Kernel, etc., used to convert lower-dimensional data into higher-dimensional data to help in the search for a hyperplane.
3. Boundary Lines: These are the two lines drawn around the hyperplane at a distance of ε (epsilon). They are used to create a margin between the data points.
4. Support Vectors: These are the data points that are closest to the boundary, which helps in defining the hyperplane.

In this research, several trials were performed to find the best model with different folds. It was found that using 8-fold cross-validation (K = 8), as illustrated in Figure 4B, gave the best performance for all SVR models, with 264 training data points and a fold size of 33 records (cases) per fold. Thus, the training data was initially divided into eight folds (K = 8). For each iteration of the eight iterations: A model was developed from the seven training folds. The model was then used to calculate the prediction for each record in the test fold (validating fold). These predictions were then used to calculate the accuracy measure.

This process is repeated for each iteration. In the end, the overall accuracy was determined by averaging the accuracy measures from the eight iterations, and the model with the best performance (with the slightest error) was chosen. After training and optimization to achieve the optimal combinations of model hyperparameters, the developed models of (Traffic, PC, and HV mean FFS) were evaluated by the testing dataset.

The “Regression Learner” app was used in MATLAB to implement the SVR model (Regression Learner App, 2021). SVR models with the quadratic polynomial kernel gave the best performance. To evaluate the performance, MSE, which was defined earlier in Eq. (4) along with RMSE and MAE metrics, was used and defined as follows:

(10) $$MAE=\frac{1}{n}\sum_{i=1}^{n}\left|y_{\left(act\right)i}-y_{\left(pred\right)i}\right|$$

(11) $$RMSE=\sqrt{MSE}=\sqrt{\frac{1}{n}\sum_{i=1}^{n}{\left(y_{\left(act\right)i}-y_{\left(pred\right)i}\right)}^{2}}$$

(12) $$K\left(X,X_{i}\right)={\left[\gamma *\left(X.X_{i}\right)+coef\right]}^{d}$$

Where K in Eq. (12) is the kernel function, d is the polynomial degree, e.g., d = 2 for quadratic, *γ* is a kernel parameter. All three parameters (γ, coef, d) affect the complexity and performance of the model (Ren et al., 2016). Table 6 (and Appendix, Figures A.9 to A.11) below shows the model validation summary after the training was done.

**Table 6:** SVM summary.

	RMSE		
**Mean FFS Models**	**Traffic**	**PC**	**HV**
Linear SVM	2.8402	2.9741	4.3232
Quadratic SVM	1.7414	1.807	3.3858
Cubic SVM	1.8891	2.0115	3.562
Fine Gaussian SVM	4.7136	4.6941	4.9921
Medium Gaussian SVM	1.865	1.8534	3.4101
Coarse Gaussian SVM	3.3493	3.2518	4.6376
**Best Model (Validation Summary)**	**Quadratic SVM**	**Quadratic SVM**	**Quadratic SVM**
RMSE	1.7414	1.807	3.3858
MSE	3.0324	3.2651	11.463
MAE	1.3398	1.393	2.7017
R^2^	0.88	0.87	0.58

As shown in Table 6, many SVR models were developed using the cross-validation technique. SVR models with the quadratic polynomial kernel were found to give the best performance in terms of RMSE of 1.7414, 1.807, 3.3858, and R^2^ of 0.88, 0.87, 0.58 for traffic, PC, and HV, respectively. Moreover, although R^2^ for HV was improved, more individual speed data are still needed to give better performance.

#### 4.2.3 Random Forest (RF)

**Figure 5:**
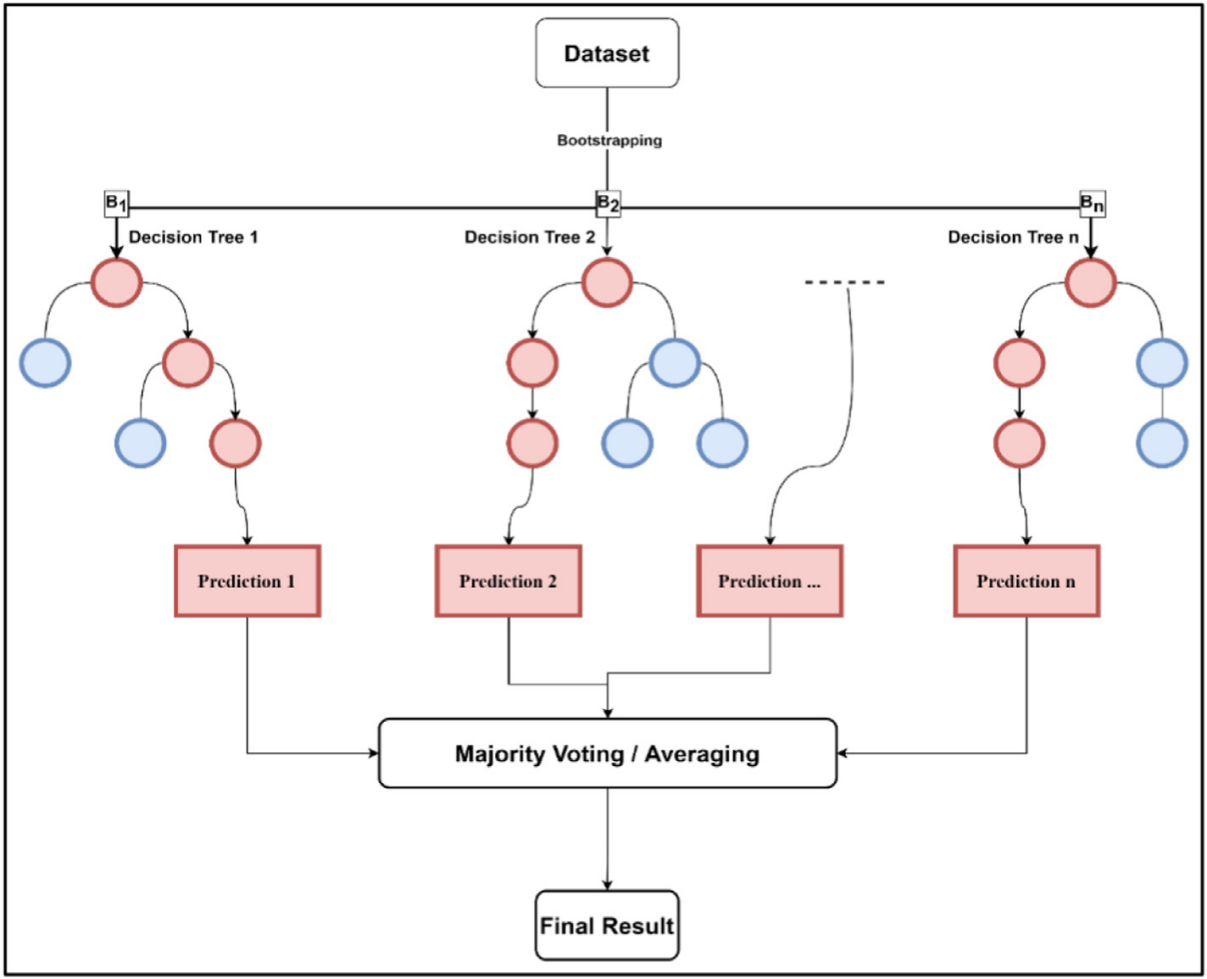
RF architecture.

Random Forest is a supervised learning technique that solves regression and classification problems using ensemble learning approaches (bagging). As demonstrated in Figure 5, the method works by constructing a large number of decision trees during the training process and then calculating the mean (averaging of results in regression problems) or mode (majority vote of results in classification problems) of the individual trees' predictions (Abdulkareem et al., 2021).

Even though decision trees are an important part of the RF algorithm and an effective regression and classification model (Nisbet et al., 2018), few drawbacks can obstruct their efficient implementation. One major issue is that decision trees are extremely sensitive to the data they are trained on, making them susceptible to overfitting; Small changes to the training set can result in significantly different tree structures. As a result, the model will make erroneous predictions on samples that have not yet been seen (test data). The RF comes to solve such an issue; There is no interaction between individual trees in a RF since each tree is built using specific random predictors, and the optimal split at each node is determined using those random variables rather than choosing the best split among all predictors. RF is an prediction algorithm that ensembles the results of several decision trees to produce the best possible outcome.

Ensemble learning is a technique that combines the predictions of numerous ML algorithms to provide more accurate predictions than any single model could produce on its own. An ensemble model, to put it simply, is one that is made up of several models. Many ensemble approaches exist, including bagging, stacking, and blending.

In this research, numerous trials with various folds were conducted to obtain the optimal model. It was discovered that utilizing 11-fold cross-validation (K = 11) achieved the best performance for all RF models. So, as a first step, the training data were split into 11 folds (K = 11). For each of the 11 iterations, the ten training folds were bootstrapped to generate 30 bootstraps resample (B = 30). For each bootstrap resample (B); A regression tree is fully grown (with the use of a random subsample of variables or features to split on at each node). The prediction for each case (record) in the test fold was then obtained by bagging the predictions from all the trees. The accuracy measure was then computed from these predictions.

This process is repeated for each iteration. In the end, the overall accuracy was determined by averaging the accuracy measures from the 11 iterations, and the model with the best performance (with the smallest error) was chosen. After training and optimization to get the best combinations of the model hyperparameters, the developed models of (Traffic, PC, and HV mean FFS) were then evaluated by the testing dataset.

The MATLAB “Regression Learner” package (Regression Learner App, 2021) implemented the RF and regression tree models. In order to evaluate the performance, MSE, RMSE, and MAE metrics were used, which were defined earlier in Eqs. 4, 10, and 11, respectively. Table 7 below (and Appendix, Figures A.12 to A.14) shows the model validation summary after the training was done.

**Table 7:** RF summary.

	RMSE					
**Mean FFS Models**	**Traffic**		**PC**		**HV**	

RF	1.8805		1.9797		2.9937	
Fine Tree	2.0846		2.1927		3.5204	
Medium Tree	2.1639		2.052		3.3175	
Coarse Tree	3.0155		3.0904		4.1439	

**Best Model (Validation Summary)**	**RF**	**Fine Tree**	**RF**	**Medium Tree**	**RF**	**Medium Tree**
RMSE	1.8805	2.0846	1.9797	2.052	2.9937	3.3175
MSE	3.0324	4.3454	3.9192	4.2106	8.9625	11.006
MAE	1.3398	1.5411	1.4911	1.511	2.3936	2.6576
R^2^	0.86	0.82	0.84	0.83	0.67	0.59

As seen in Table 7, many decision tree models were obtained, and RF models were developed using cross-validation combined with the bootstrapping technique. Bagged RF models were found to give the best performance in RMSE of 1.8805, 1.9797, 2.9937, and R^2^ of 0.86, 0.84, 0.67 for traffic, PC, and HV, respectively. It can be seen that R^2^ for HV was improved in RF and gives better performance than SVR, which suggests that RF has an excellent capability to deal with small and noisy data.

### 4.3 Models evaluation

**Figure 6:**
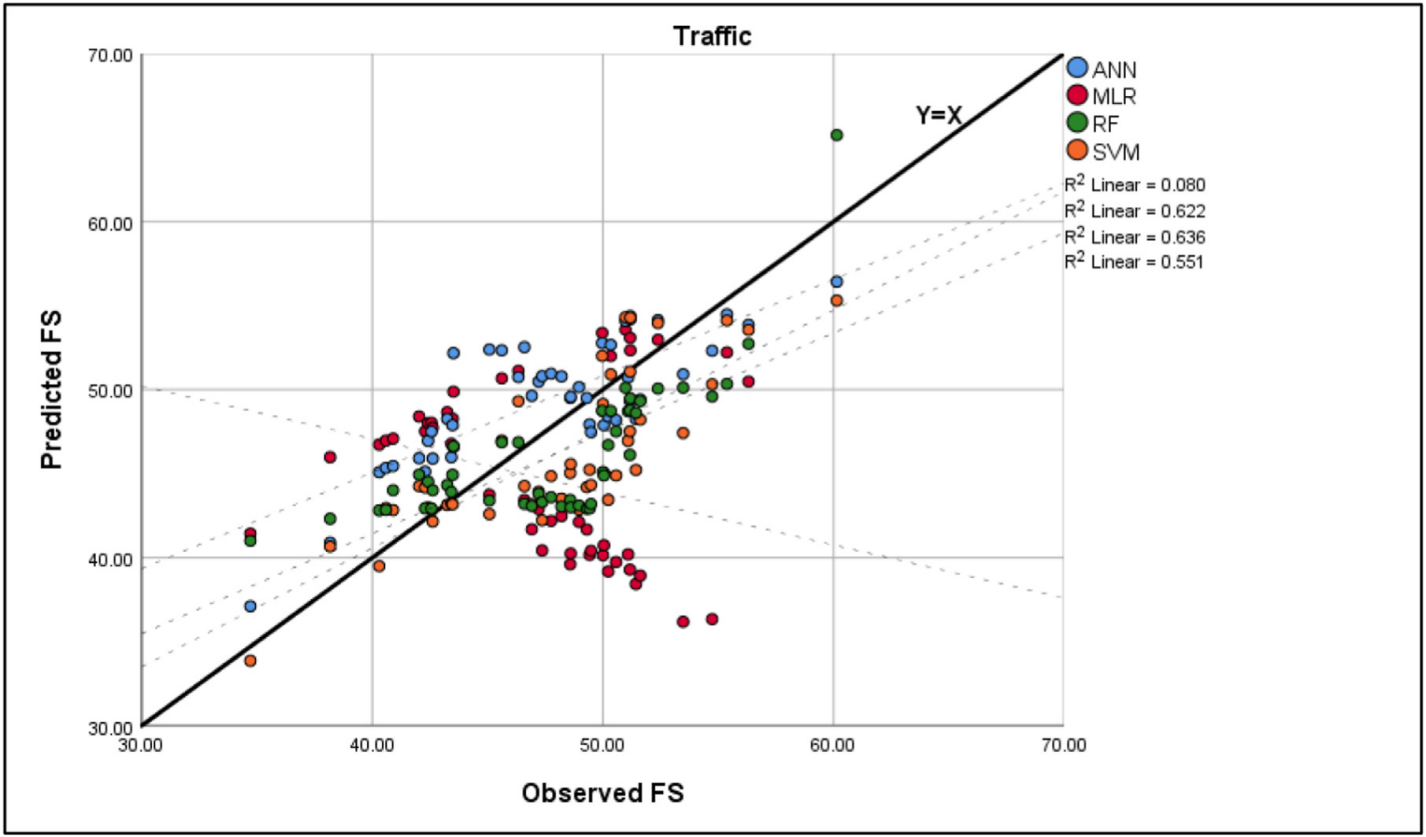
Observed and predicted FS for traffic.

**Figure 7:**
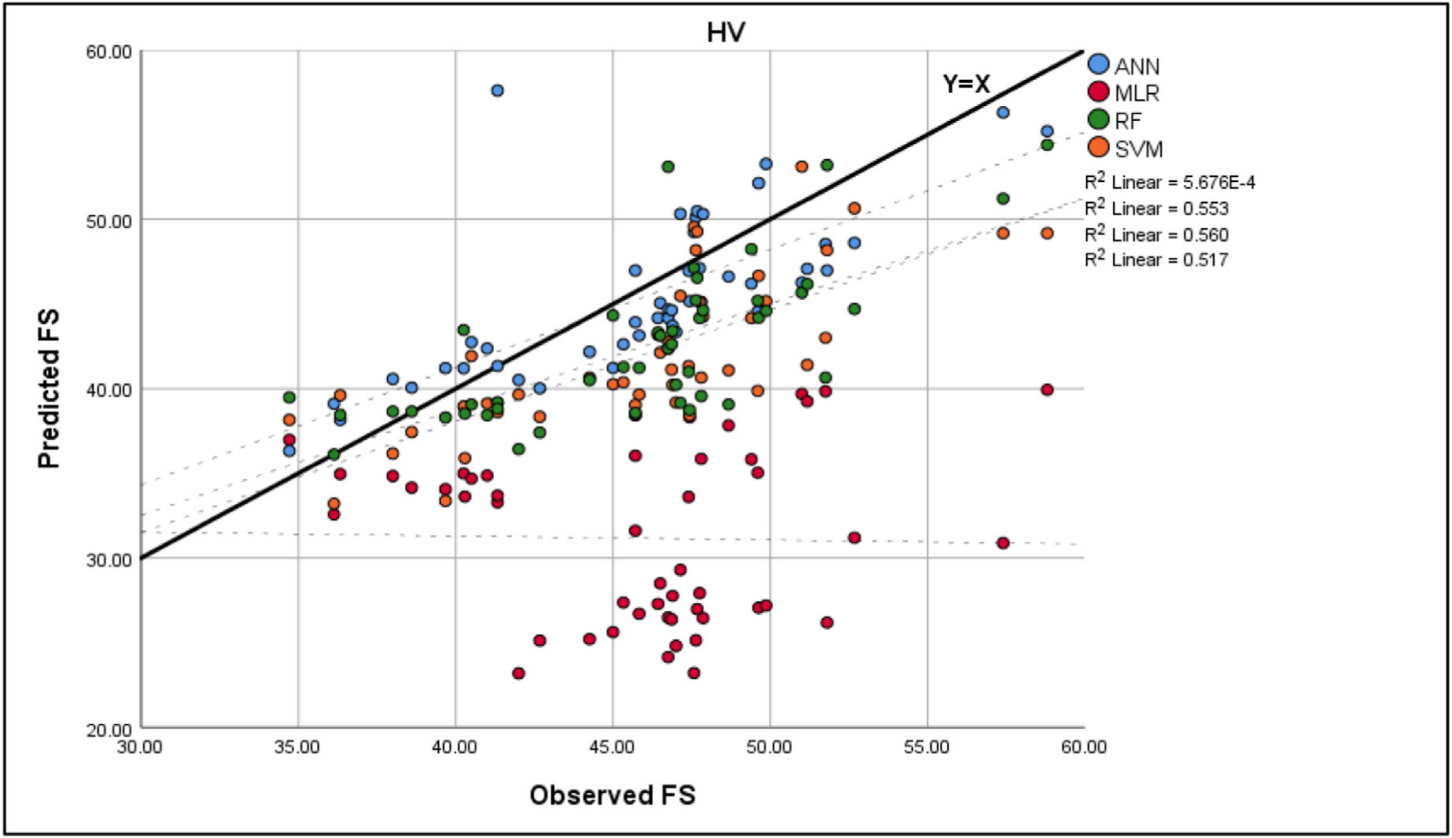
Observed and predicted FS for PC.

**Figure 8:**
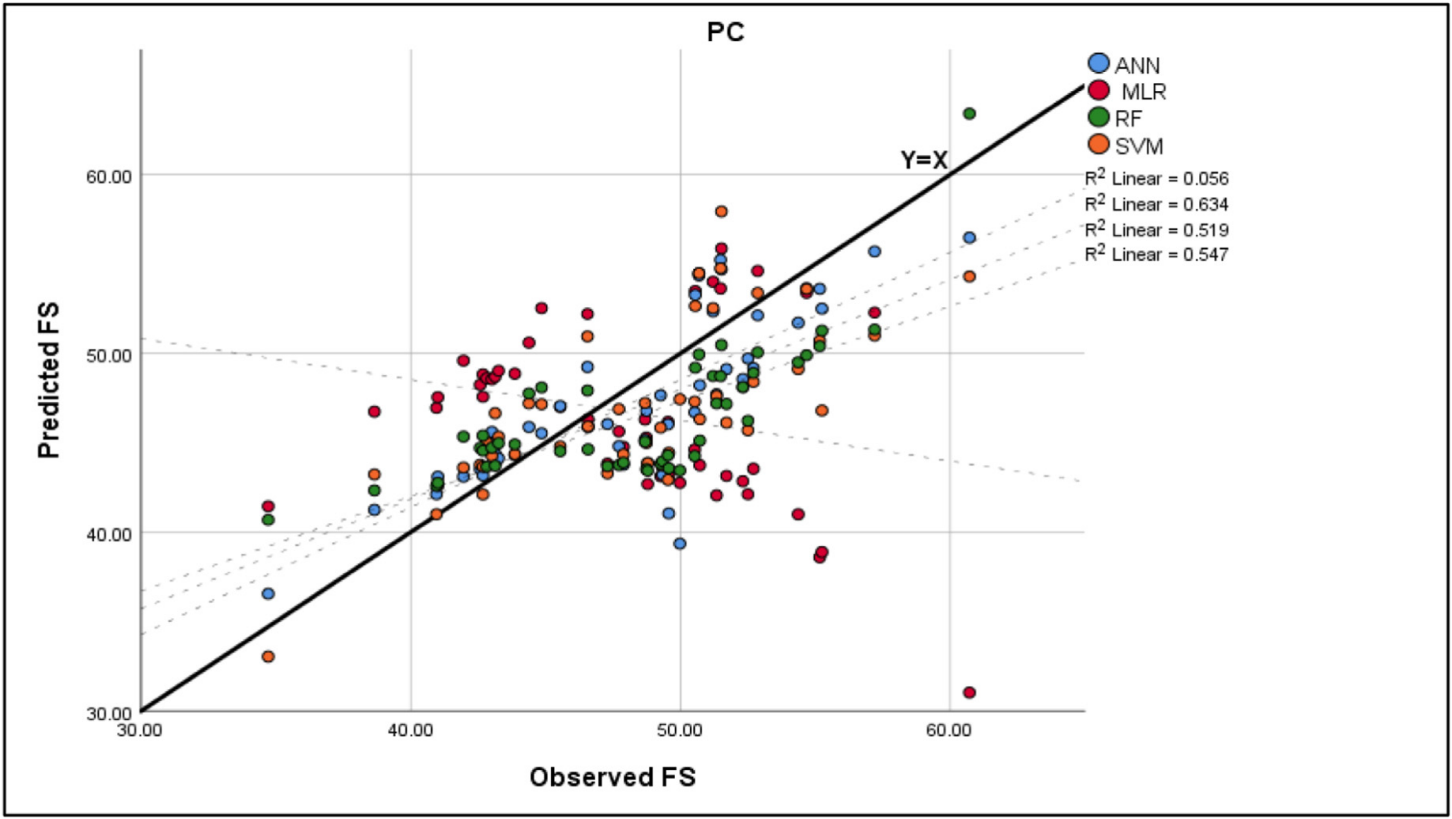
Observed and predicted FS for HV.

**Figure 9:**
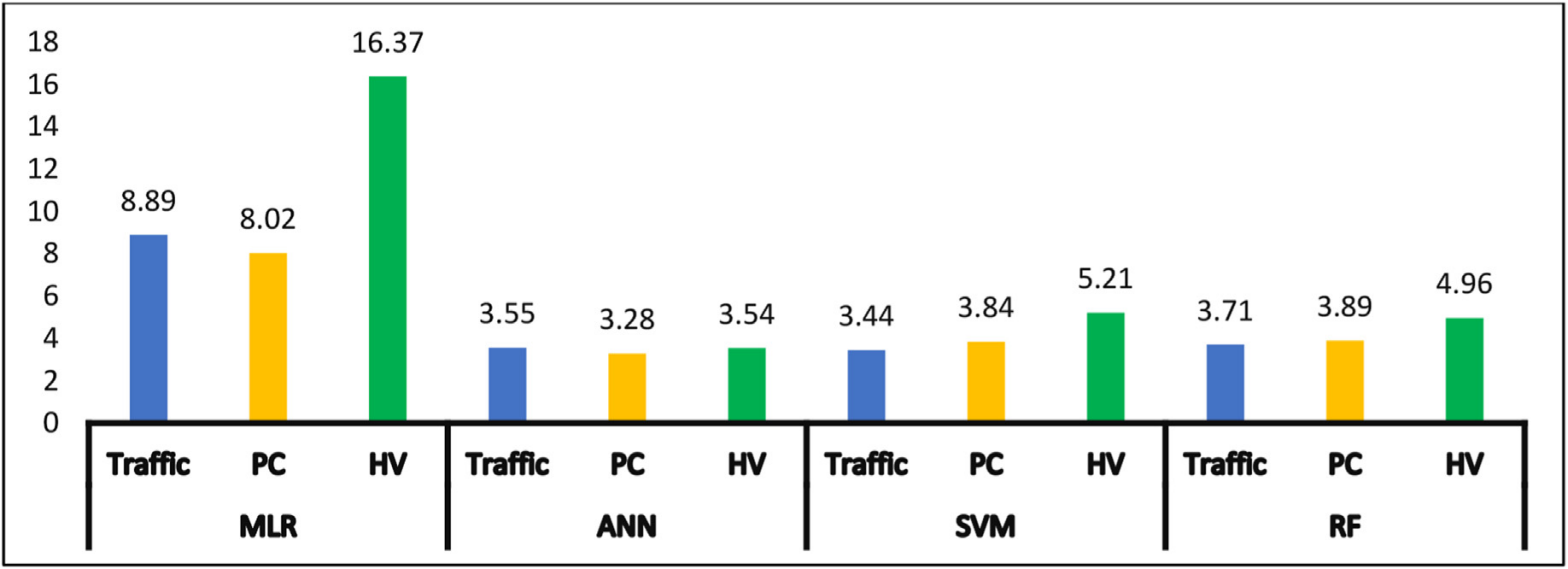
RMSE for test data (evaluation summary).

In order to evaluate the performance of each model after the training and validation processes, test data was collected later on from two randomly selected roads that were not used in the training stage for 2 h in each direction, with 12 intervals per direction, each equal to 10 min in duration, resulting in 48 overall study intervals, then the average speed was calculated in each interval to produce 48 data points that were used as a testing data set. This evaluation involves a comparison between the prediction and actual results. The best fit is presented in Figures 6, 7, 8, 9 for the observed and predicted mean FFS. R^2^ was used to evaluate the goodness of fit between the observed and predicted values. RMSE was used to evaluate the prediction performance for each model. Table 8 shows the model evaluation summary using the test data.

**Table 8:** Model evaluation summary.

Models		RMSE	R^2^
MLR	Traffic	8.889349	0.08
	PC	8.023939	Too small
	HV	16.36648	Too small
ANN	Traffic	3.552852	0.622
	PC	3.27535	0.634
	HV	3.538895	0.553
SVM	Traffic	3.442077	0.636
	PC	3.837612	0.519
	HV	5.20526	0.56
RF	Traffic	3.71069	0.551
	PC	3.892805	0.547
	HV	4.960306	0.517

The highest value of the RMSE of the MLR model, especially in the HV mean FFS case, suggests that the errors are more scattered than in ML models, which were applied in this study. Also, the outliers were not removed from the test data, resulting in a high value of RMSE since RMSE is sensitive to outliers. Furthermore, the distribution of those red dots indicates that MLR could not explain the variability or variance of the test sections; thus, R^2^ was very small in all cases.

Moreover, as shown in Table 8, the comparison results indicate that the outputs of ML models had less prediction error than regression models. Also, the coefficients of determination R^2^ between observed and predicted free speed (FS) values of ML models were significantly higher than regression models. These results indicate that the developed ML models gave more accurate results than the regression approach in the training or testing process.

Lastly, it is observed from Figure 9 and Table 8 that the RMSE of the test sections for ANN models is approximately the same and has the lowest values for all types of traffic, which suggests that the errors are more concentrated around the line of equality (Y = X) and there are fewer minor errors as compared to the other models. In terms of goodness of fit, the R^2^ of ANN was found to be the highest among other ML models, except in the traffic SVM model, which was equal to 0.636. Still, the difference between these two models is not considerable (0.014).

### 4.4 Comparison between the results obtained in present study with previous studies

The following Table 9 summarizes the results obtained from previous studies. Only the models with the greatest performance were introduced.

**Table 9:** Comparison between the results obtained in present study with previous studies.

Author	Prediction Technique	Dependent variable	Independent Variables {Variables} = Significant	R^2^	Performance Metric
Maczyński et al. (2021)	MLR	Light Vehicles FFS	{Percentage of Vehicles, Lane Width, Shoulder Width, Type of Day} Average Hourly Traffic, Time	0.469	Mean value of predicted errors: 3.6 km/h
		Heavy Vehicles FFS	{Percentage of Vehicles, Shoulder Width, Type of Day} Average Hourly Traffic, Lane Width, Time	0.251	2.6 km/h
	ANN	Light Vehicles FFS	Same Inputs		2.5 km/h
		Heavy Vehicles FFS			1.9 km/h
Alomari et al. (2021a, b)	MLR	Speed Variance	{Number of Lanes, Presence of Roadside Barriers, Design Speed - Speed Limit} Lane Width, Design Speed, Speed Limit	0.835	SSE = 13.59
	Nonlinear (Quadratic)	Speed Variance	{Design Speed - Speed Limit}	0.697	SEE = 18.452
Bratsas et al. (2020)	MLR	Mean Speed	{Minimum, Maximum, Standard Deviation, Skewness, Kurtosis of Speed, Car Entries, Unique Car Entries}		MAE = 6.90
	ANN				MAE = 6.57
	SVR				MAE = 6.25
	RF				MAE = 6.44
Rao and Rao (2015)	MLR	FFS	{Total Vehicles, Number of Friction Points, Major Intersections, Number of Flyovers, Access Points, Section Length}	0.541	SEE = 3.372
Semeida (2013)	MLR	V_85_	{Right Shoulder Width, Existence of Side Access, Posted Speed Limit} Lane Width, Pavement Width, No. of Lanes, Median Width	0.761	RMSE = 10.32
	ANN	V_85_	Same Inputs	0.978	RMSE = 3.11
Singh et al. (2011)	ANN	V_85_ with PS	{Surface Width, Shoulder Type, Shoulder Width, Average Daily Traffic, Skid Number, IRI, Posted Speed}		MARE = 3.1%
		V_85_ without PS	Same Inputs but Without Posted Speed		MARE = 8.6%

According to Table 9, the following can be noted:

• **For MLR**

Lane width was found to have a significant positive impact on vehicular speed in (Maczyński et al., 2021) study, which agrees with the current study. However, lane width was not significant in (Semeida, 2013; Alomari et al., 2021a, b) studies. Median width had a significant negative influence in the present study but was not significant in Semeida (2013) study. The number of lanes was reported to have a significant negative effect on vehicular speed variance, which implies that when the number of lanes increases, the vehicle can maintain a relatively high speed, so the speed variance will decrease, which is consistent with our findings. On the other hand, the number of lanes was insignificant in (Semeida, 2013) findings.

The number of access points was found to have a significant positive impact in the present study, which agrees with (Rao and Rao, 2015). However, it was had a negative impact on the vehicular speed on Egyptian highways (Semeida, 2013). However, it should be emphasized that this variable was not influential in earlier studies conducted outside of Egypt. Traffic volume was found to have a negligible positive impact (β = 0.007) in (Rao and Rao, 2015) but has a negative impact in the present study. In contrast, traffic volume was insignificant in (Maczyński et al., 2021) research.

In terms of speed as an independent factor, Alomari et al. (2021a, b) found the difference between design speed and speed limit would increase the speed variance when the difference is more than 10 km/h. This is most likely because, regardless of the speed limit, drivers tend to operate their vehicles based on road conditions, which are governed by the design speed (i.e., high vehicular speed). Moreover, Semeida (2013) found a significant positive influence between posted and traffic speed. On the other hand, this study considered the absolute difference between the speed limit and 85^th^ percentile, and the effect of this variable was deeply discussed previously.

• **For Machine Learning Models**

Many studies; Singh et al. (2011); Semeida (2013); Bratsas et al. (2020); Maczyński et al. (2021), considered ANN as an analysis technique due to its reliable performance capability to predict traffic speed with lower prediction error as indicated in the performance measure in the last column of Table 9. In this study, ANN also provided the best performance across other techniques. Moreover, Bratsas et al. (2020) have considered SVM and RF. Both gave good results, and this was consistent with the current study in which SVM and RF were found as suitable alternatives when the amount of data is less or noisy in nature, such as the HV case. This finding was also supported in another research (Vanajakshi and Rilett, 2007).

## 5 Conclusions

The present study developed MLR and ML models, including ANN, SVM, and RF, to predict the mean FFS using several independent variables. A comprehensive literature review was conducted first. The target variable was the mean FFS. While geometric, traffic, and pavement condition parameters were presented as predictor variables. The traffic features group includes spot speed, speed limit, average speed, 85^th^ percentile speed, traffic and crossing pedestrian volumes, volume of exiting vehicles, Elderly%, HV%, and TCMs. The geometric characteristics include lateral clearance, number of lanes, number of access points (including median openings), road grade, effective lane width, and median width. The pavement condition category includes pavement roughness in terms of IRI. Models for each traffic composition were built (i.e., PC, HV, and overall combined traffic). Eleven urban streets were used to develop the MLR model and train the ML models. The MLR model was built using SPSS software, while the codes for the ML models were developed using MATLAB software Version R2021. Many trials and iterations were performed to achieve the optimal ML model performance. As a result, the best models' performances were shown and demonstrated.

In ANN, the best architecture was found to contain one hidden layer with ten neurons for all types of traffic. All developed models provided an overall performance coefficient of correlation (R) of above 0.9. The traffic model exhibited an overall performance of R^2^ equal to 0.96322, 0.95697, and 0.831030 for traffic, PC, and HV, respectively. In SVM, several models were developed using the cross-validation technique, and models with the second-degree polynomial kernel (quadratic) were found to give the best performance in terms of RMSE of 1.7414, 1.807, 3.3858, and R^2^ of 0.88, 0.87, and 0.58 for traffic, PC, and HV, respectively. In RF, many decision tree models were obtained, along with RF models that were developed using cross-validation combined with the bootstrapping technique. Bagged RF models were found to give the best performance in RMSE of 1.8805, 1.9797, and 2.9937, and an R^2^ of 0.86, 0.84, and 0.67 for traffic, PC, and HV, respectively. Finally, a comparison analysis was performed to evaluate the performance of each model and investigate the differences between traditional and ML techniques using test data collected later on from randomly selected roads. Moreover, based on the results of the two techniques, the best prediction models were determined.

Theoretically, in predicting mean FFS, the proposed ML algorithms outperformed linear regression models and are believed to be valuable and strong tools that adapt to sudden changes in traffic flow caused by exogenous conditions on urban arterials. However, ML algorithms cannot explain any phenomenon or behavior on the street since they do not show any statistical equation or relation. On the other hand, the MLR dose for example, based on the coefficient of |V85-PS| HV variable, could be implied that the Jordanian HV drivers (especially bus drivers) are showing aggressive behavior when they usually drive above the speed limit. Different validation techniques were used, such as cross-validation and bootstrapping, which resulted in a powerful reduction of the overfitting phenomenon. ML algorithms are comparable to regression models when used to extrapolate beyond the data provided for training. They should not be expected to perform well in such a case. Accordingly, the reliability of the model may be questionable. Therefore, ML approaches are preferred when the input parameters are within the model's development range.

Practically, the results of this study can be used in two main approaches: the evaluation approach and the planning approach. Starting with the evaluation approach, a traffic engineering consultant might use either the MLR or ML algorithms to perform any assessment of the potential changes to the urban design (e.g., establishing a new commercial building, reducing the number of lanes, increasing the median width, and so on). Also, the developed models in this study can be used to evaluate the current FFS in multiple arterials, allowing for determination of the Level of Service (LOS) and building traffic simulation models. Consequently, this study recommends that local authorities and transport agencies adopt the developed models to apply them to their future duties further. While planning for a new district, town, or even a city, a collaboration work of experts from different backgrounds is essential for a proper design. The posted speed along the arterial roads is among the critical parameters that the designers might seek to find. The typical approach is to rely upon the 85th percentile speed. However, this widespread practice comes with limitations and neglecting to consider the other attributing variables can be among the primary shortages. Therefore, the developed models present a reliable and valid tool to allow the designers to set a posted speed that fits the condition of that particular arterial road. Ultimately, this research recommends that planning consultancy groups and governing agencies utilize the presented models. Finally, it is necessary to mention that the proposed models can be used in two forms. The first form can be thought to rely on the same model parameters. However, this implies that this finding can serve as an approximation with lower accuracy. This can be adopted when quick findings are needed, and experts can assess the developed model findings based on their prior experience.

The other approach is to collect another small sample to perform a model calibration for the MLR and re-training for the ML algorithms while using the obtained parameter values from this research as initial values. This approach can be used when more accuracy is required, and some period is given for the data collection and processing of the new data.

Overall, the following tasks can be achieved using the introduced ML models:

1. Predict mean FFS with higher reliability based on the roadway and traffic-related characteristics without requiring a time-consuming spot study, which is highly helpful when time and funds are limited.
2. Help improve traffic safety and in situations where preliminary, planning-level evaluations of arterial speeds are required.
3. Validate field-collected data. The suggested ML models can be used to evaluate the impact of any proposed changes or variations in roadway-related input characteristics on the mean FFS of a specific arterial in an urban area.

## 6 Recommendations

Although the ML models have excellent capabilities in prediction, additional data, such as the size of the individual data collected, is still needed to improve the model's prediction power, especially for HV. Besides, overfitting can be reduced by increasing the number of sites in the dataset and their distribution. Further research is needed to make the results more robust in terms of consideration of the road types and land use (Kadhim et al., 2020), horizontal and vertical curve elements such as sight distance, length and grade of approach and departure tangents, side slope, and radius of the horizontal curve (Taylor et al., 2007), vehicle and driver characteristics such as age and gender (McKelvey et al., 1988; Polus et al., 1991), vehicle characteristics were not considered as well. Using videotape or obtaining additional staff (observers) would help.

It is recommended to consider the acceleration and deceleration distances since they guide the determination of the minimum distance required of the intended study sites between two intersections with a traffic control device (Wang et al., 2006). In this way, it can be ensured the selected streets will be sufficiently long for drivers to choose and achieve their desired speeds without the influence of the adjacent traffic control device.

Data were gathered from sections of pavement that were in good condition generally and had not deteriorated with no sharp horizontal curves or steep slopes. As a result, the suggested models are unable to generalize using data from such sections. These sections can be used to establish other models that can be compared to the current models.

Because some traffic authorities in third-world countries, such as Jordan, lack clear rules for installing speed humps, the impact of TCMs (represented by speed humps and cat-eye reflectors) was characterized by the observer's judgment. Calibration is still needed on the collected roughness data on Jordanian roads using a smartphone, with roughness data collected using standard procedures to check the accuracy of the smartphone method (Alatoom and Obaidat, 2021). Also, utilizing map-matching algorithms is recommended to remove the GPS noise within the collected roughness data. ML models should be validated and evaluated using new data not utilized in the model's training or testing. For these models to indirectly consider any changes in traffic legislation or driver behavior, frequent re-training of the created models (with updated input and output data) is also recommended.

## Declarations

### Author contribution statement

Ahmad H. Alomari, Abdel Rahman O. Marian: Conceived and designed the experiments; Performed the experiments; Analyzed and interpreted the data; Contributed reagents, materials, analysis tools or data; Wrote the paper.

Taisir S. Khedaywi: Conceived and designed the experiments; Analyzed and interpreted the data; Wrote the paper.

Asalah A. Jadah: Performed the experiments; Contributed reagents, materials, analysis tools or data.

### Funding statement

This research did not receive any specific grant from funding agencies in the public, commercial, or not-for-profit sectors.

### Data availability statement

Data will be made available on request.

### Declaration of interests statement

The authors declare no conflict of interest.

### Additional information

No additional information is available for this paper.
