# Supplementary material for: Traffic speed prediction techniques in urban environments
Source: Heliyon. 2022 Dec 1;8(12):e11847. doi: 10.1016/j.heliyon.2022.e11847 (PMC9732136; doi:10.1016/j.heliyon.2022.e11847)
Supplement: Appendix_08-11-2022 [file mmc1.docx]

# **APPENDIX**


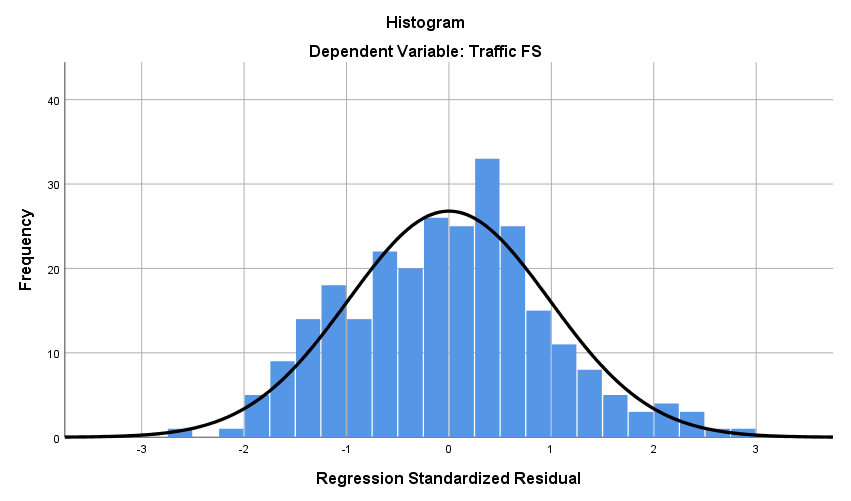

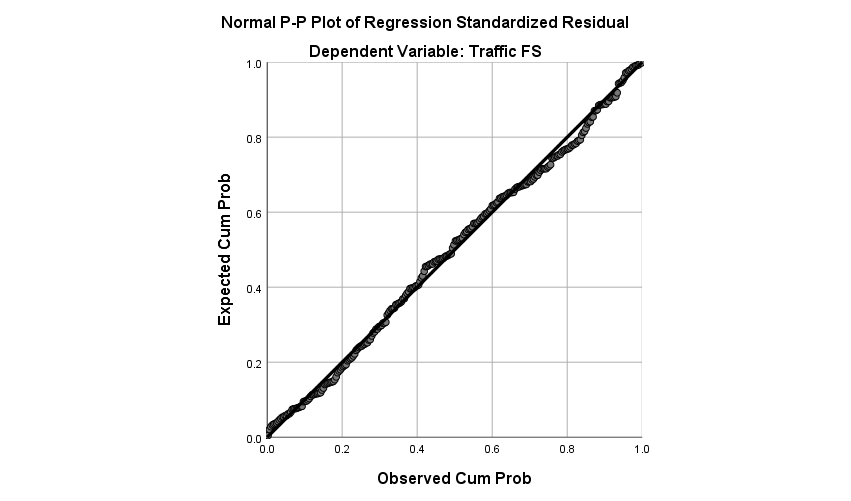

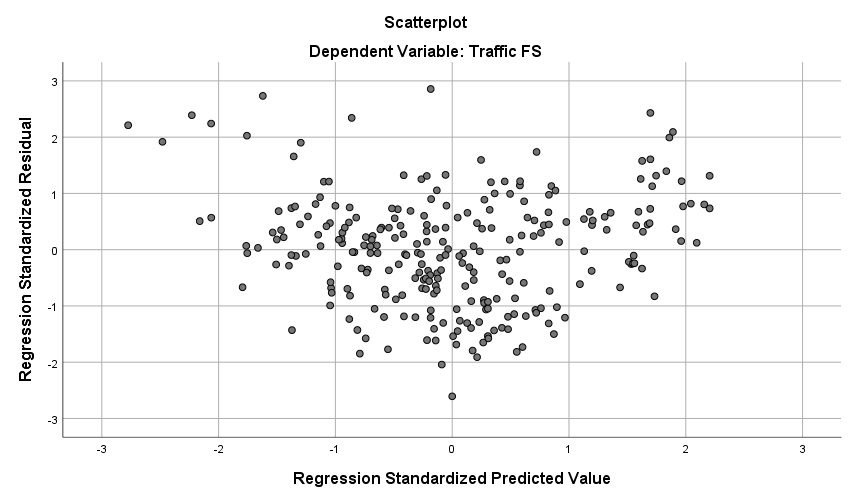


**a**

**a**

**a**

**a**

**b**

**b**

**b**

**b**

**c**

**c**

**c**

**c**

## Figure A.1 (a) Residual frequency histogram, (b) P-P plot, (c) Standardized residual vs predicted mean FS for traffic model.


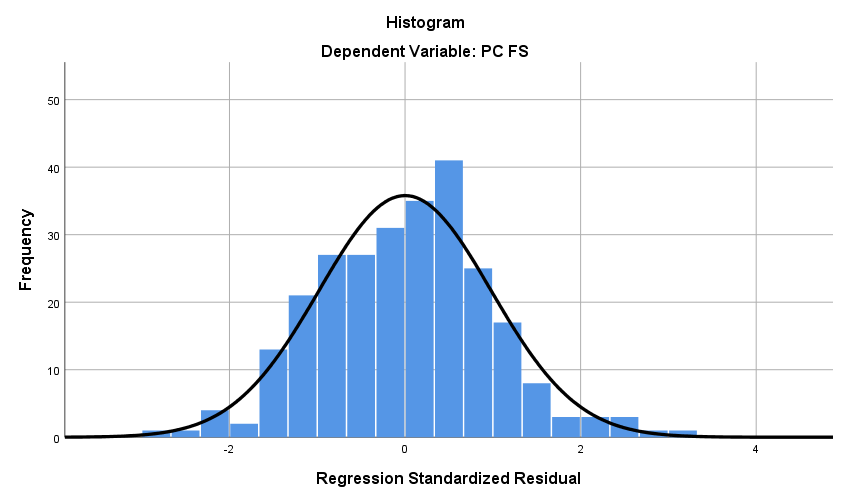

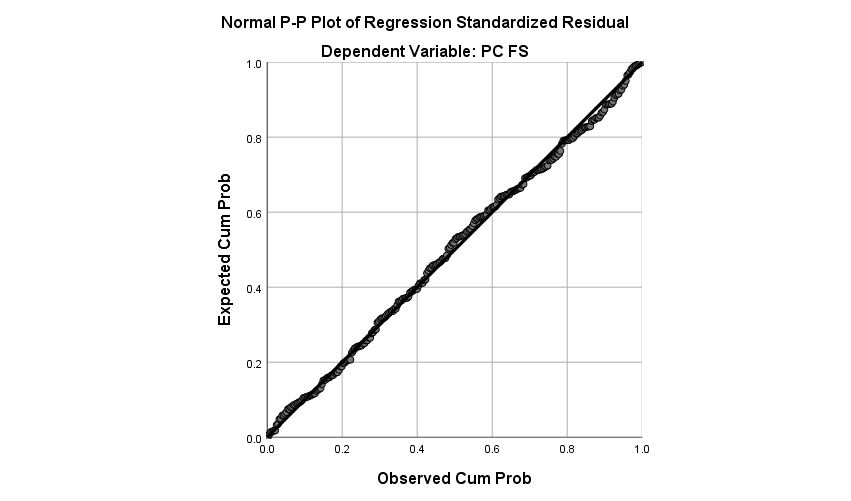

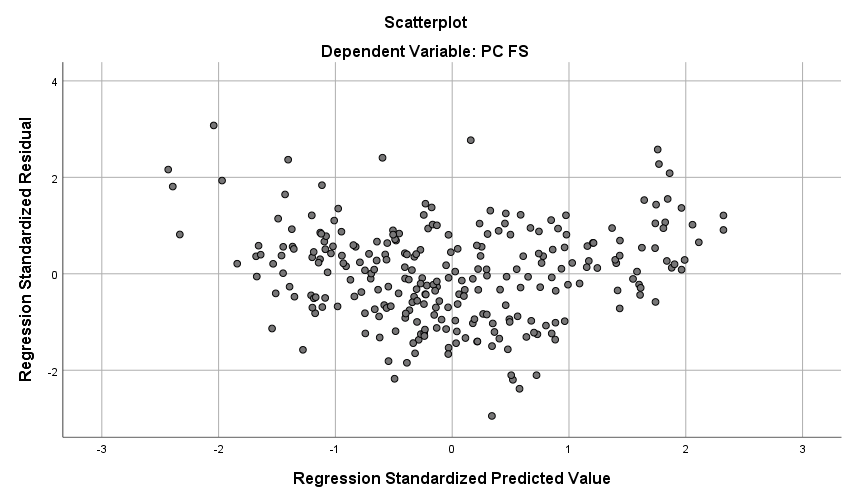


**a**

**a**

**a**

**a**

**b**

**b**

**b**

**b**

**c**

**c**

**c**

**c**

## Figure A.2 (a) Residual frequency histogram, (b) P-P plot, (c) Standardized residual vs predicted mean FS for PC model.


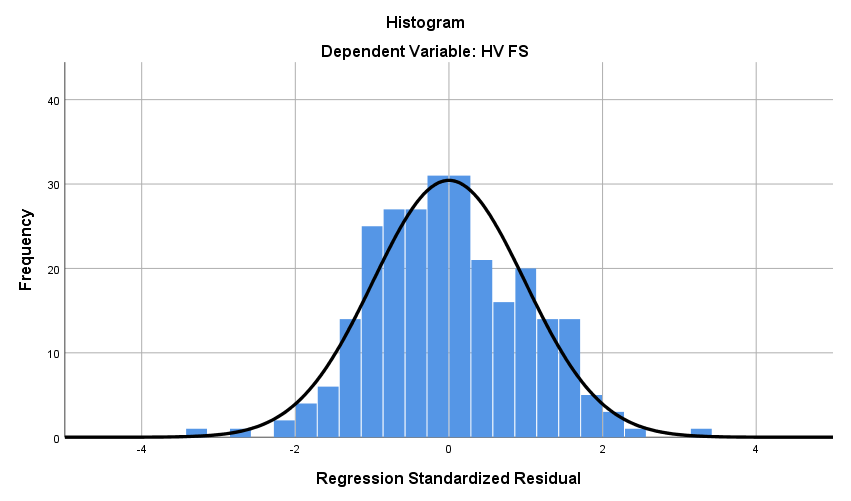

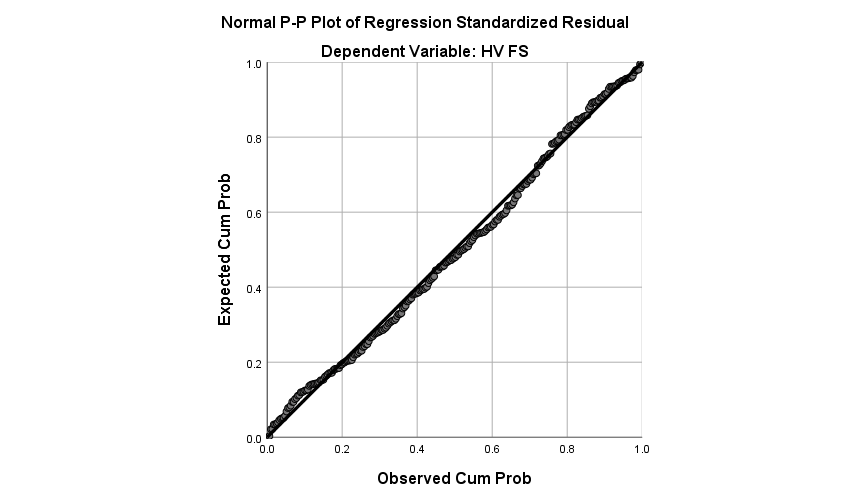

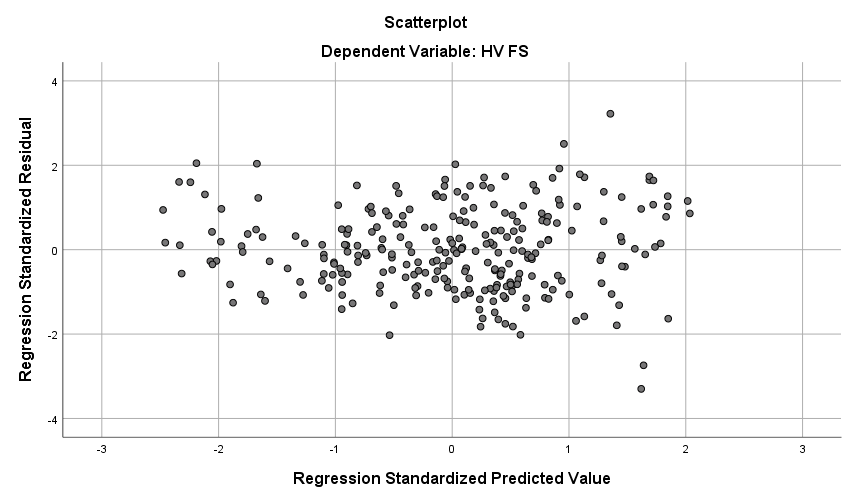


**a**

**a**

**a**

**a**

**b**

**b**

**b**

**b**

**c**

**c**

**c**

**c**

## Figure A.3 (a) Residual frequency histogram, (b) P-P plot, (c) Standardized residual vs predicted mean FS for HV model.


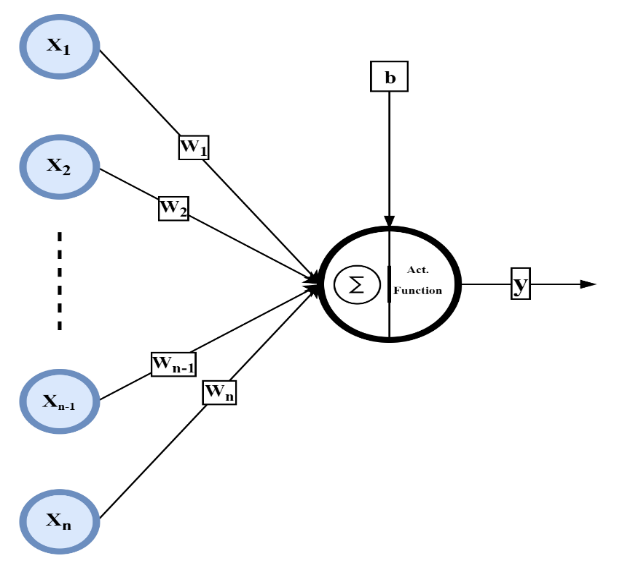


## Figure ‎A.4 Activation Function Mechanism


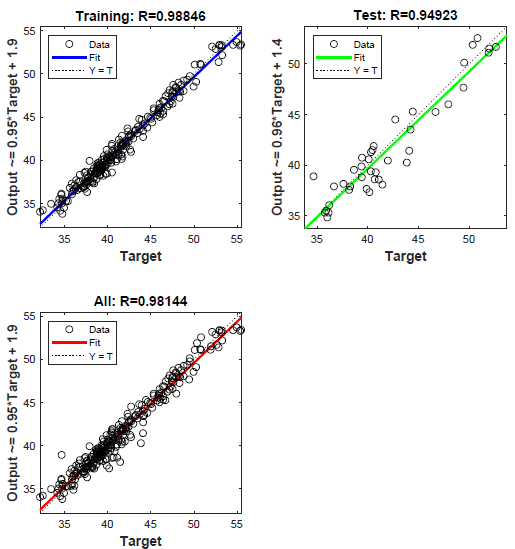


## Figure ‎A.5 Regression Plots for Traffic Free Speed Model


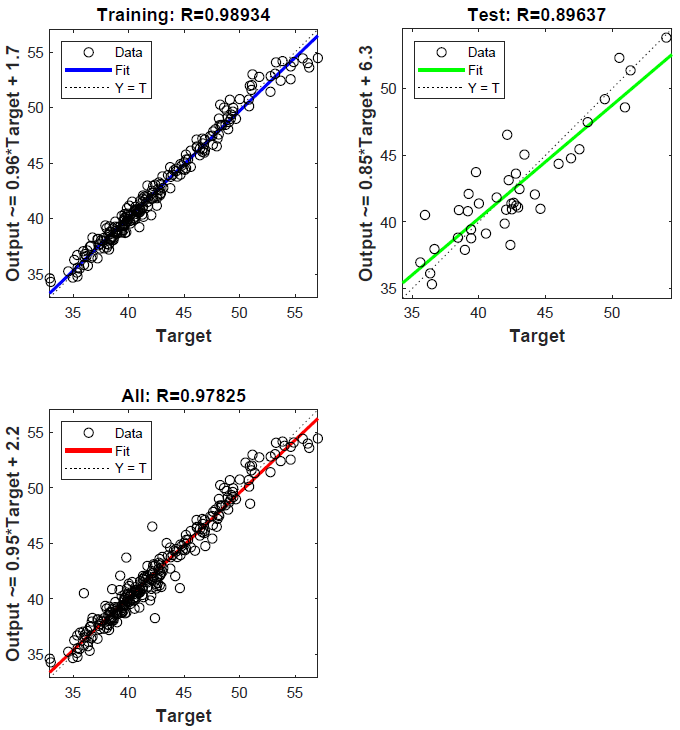


## Figure ‎A.6 Regression Plots for PC Free Speed Model


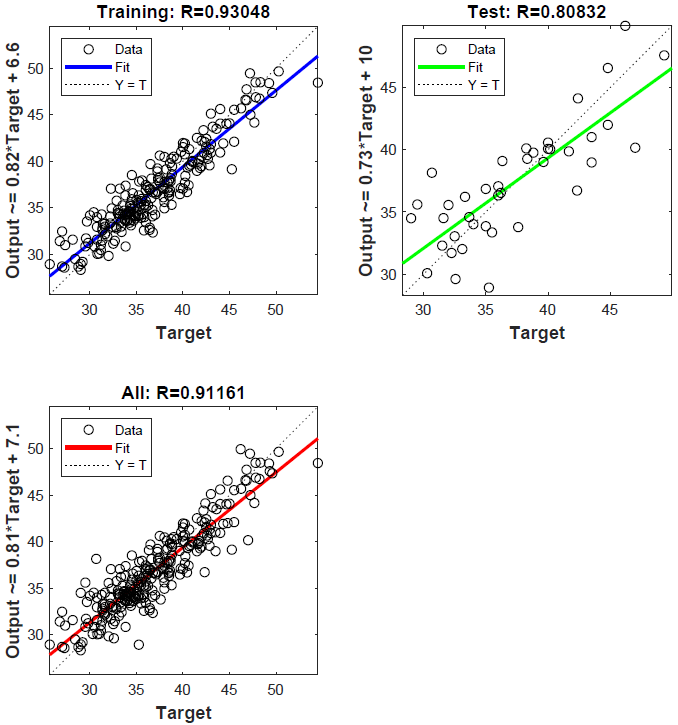


## Figure ‎A.7 Regression Plots for HV Free Speed Model


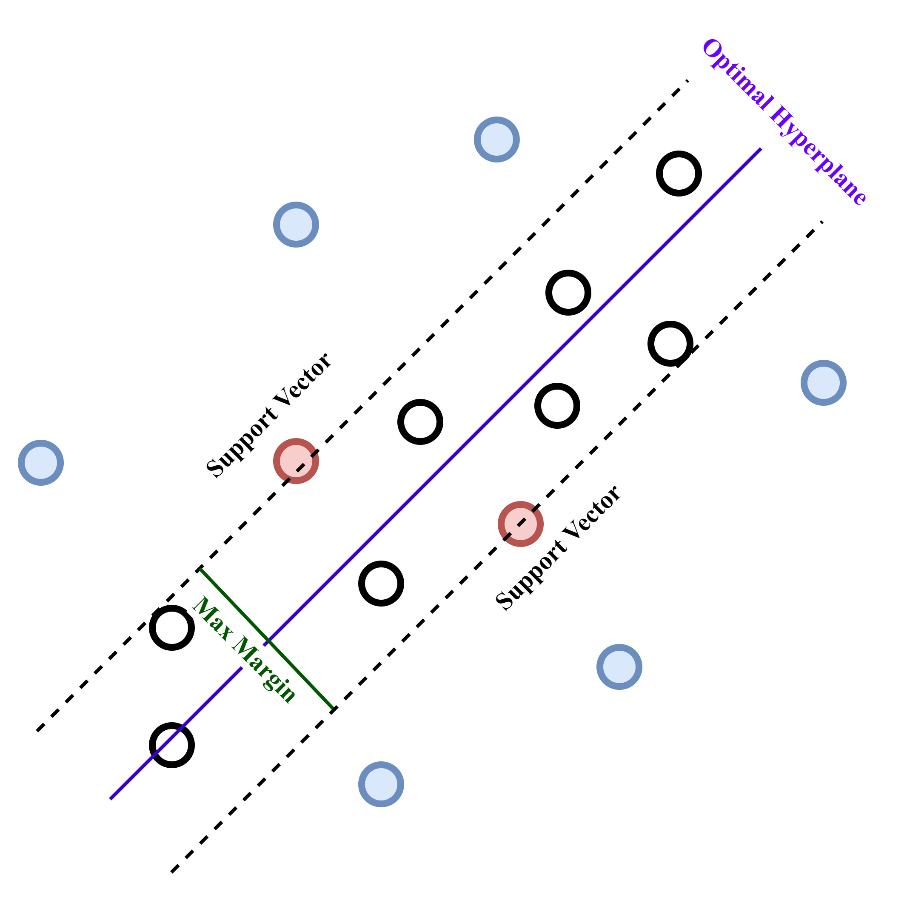


## Figure A.8 SVR Hyper-Parameters


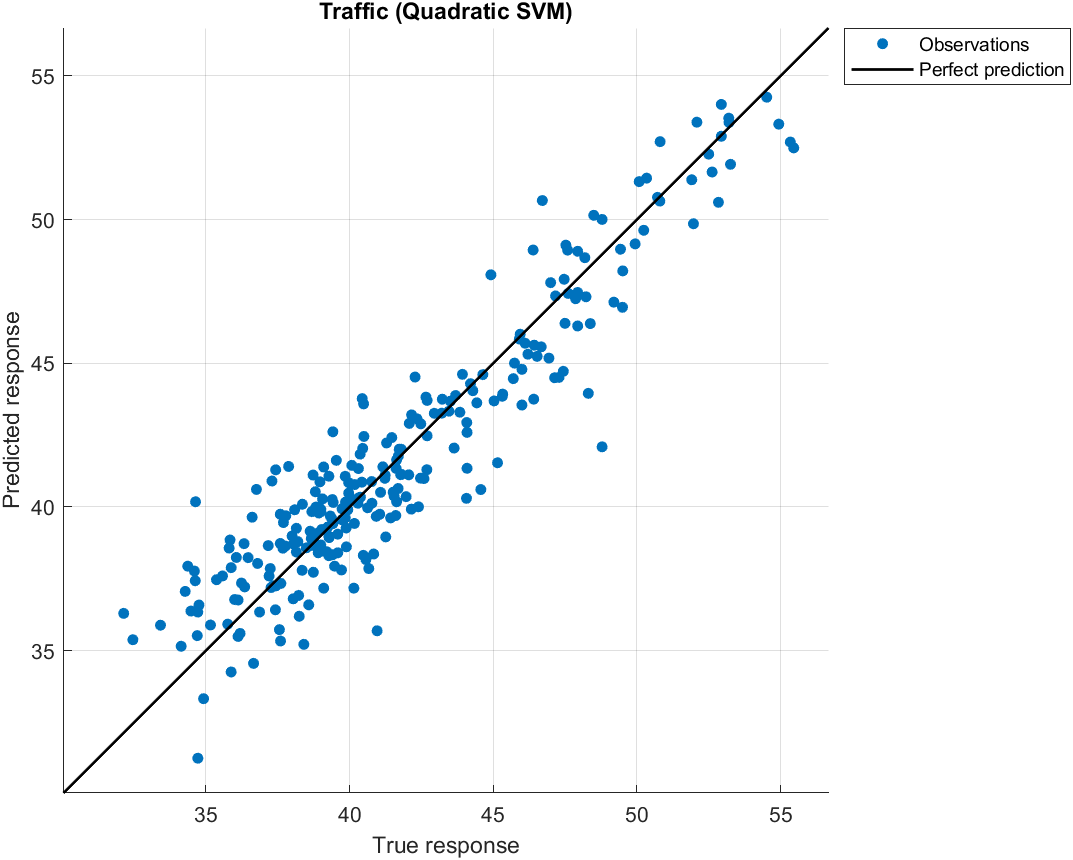

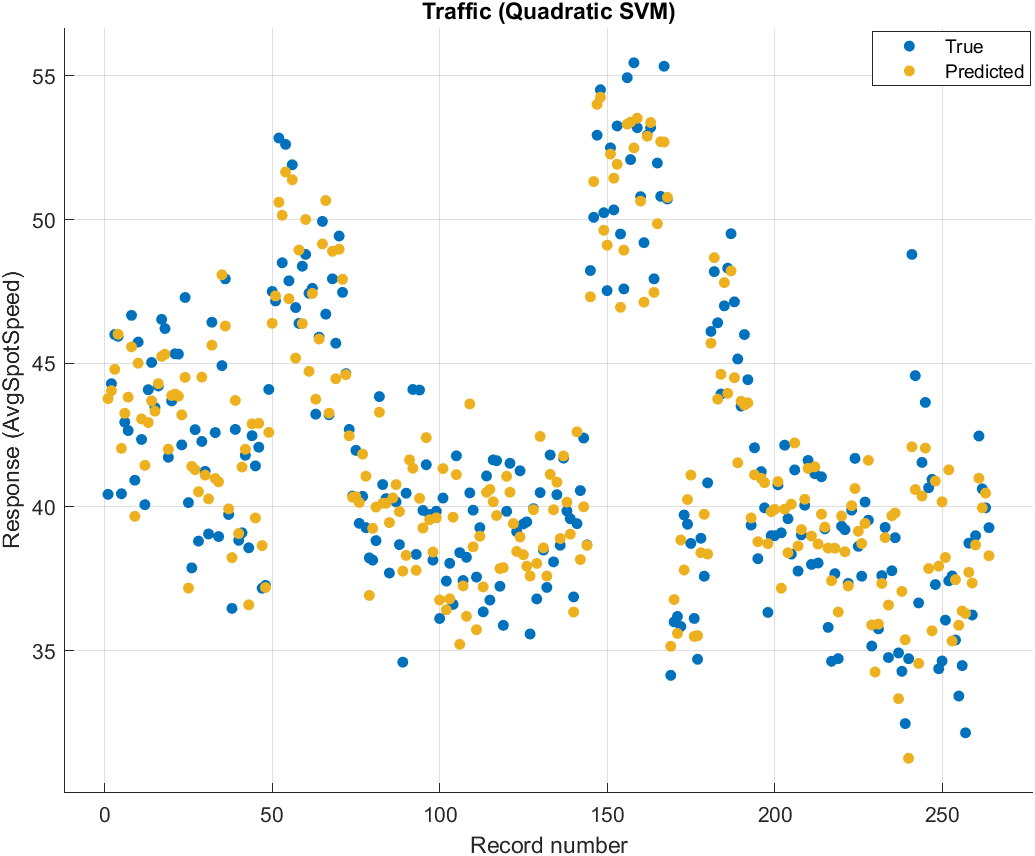

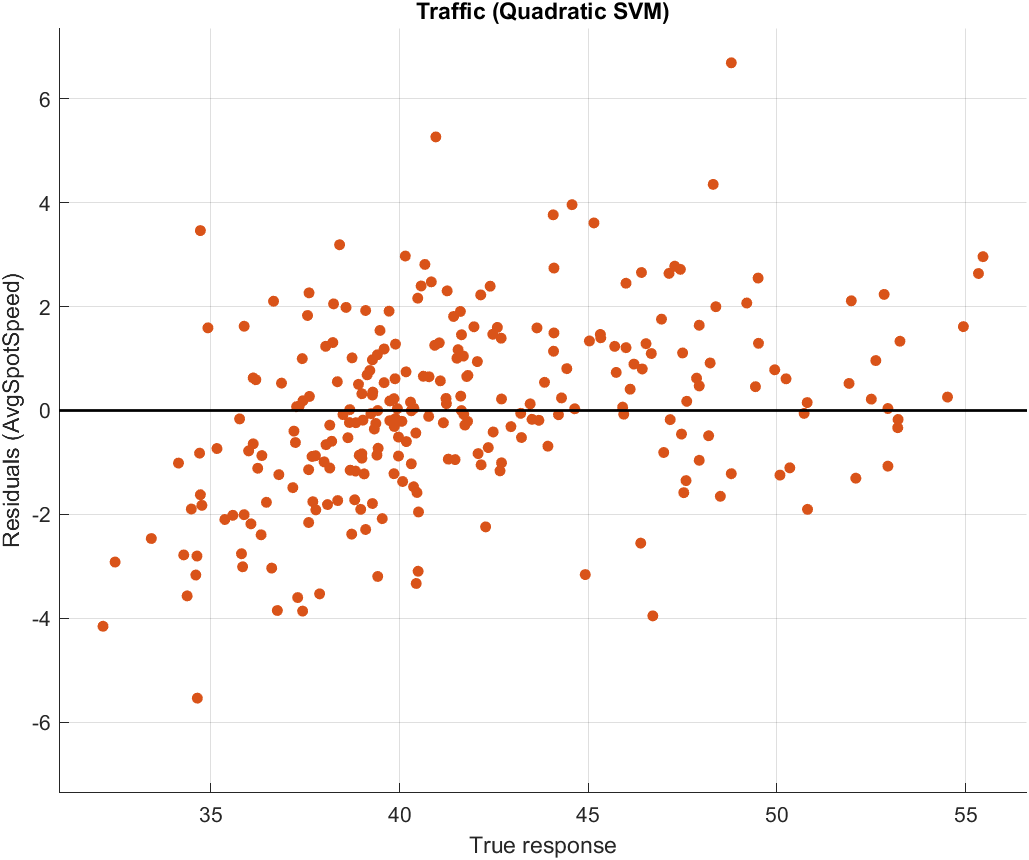


## Figure ‎A.9 Traffic SVR Summary


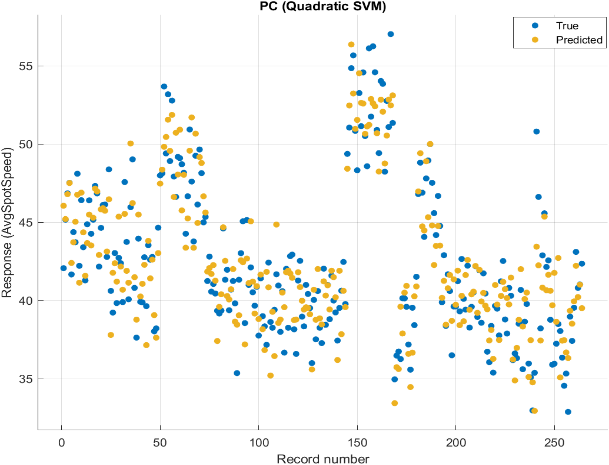

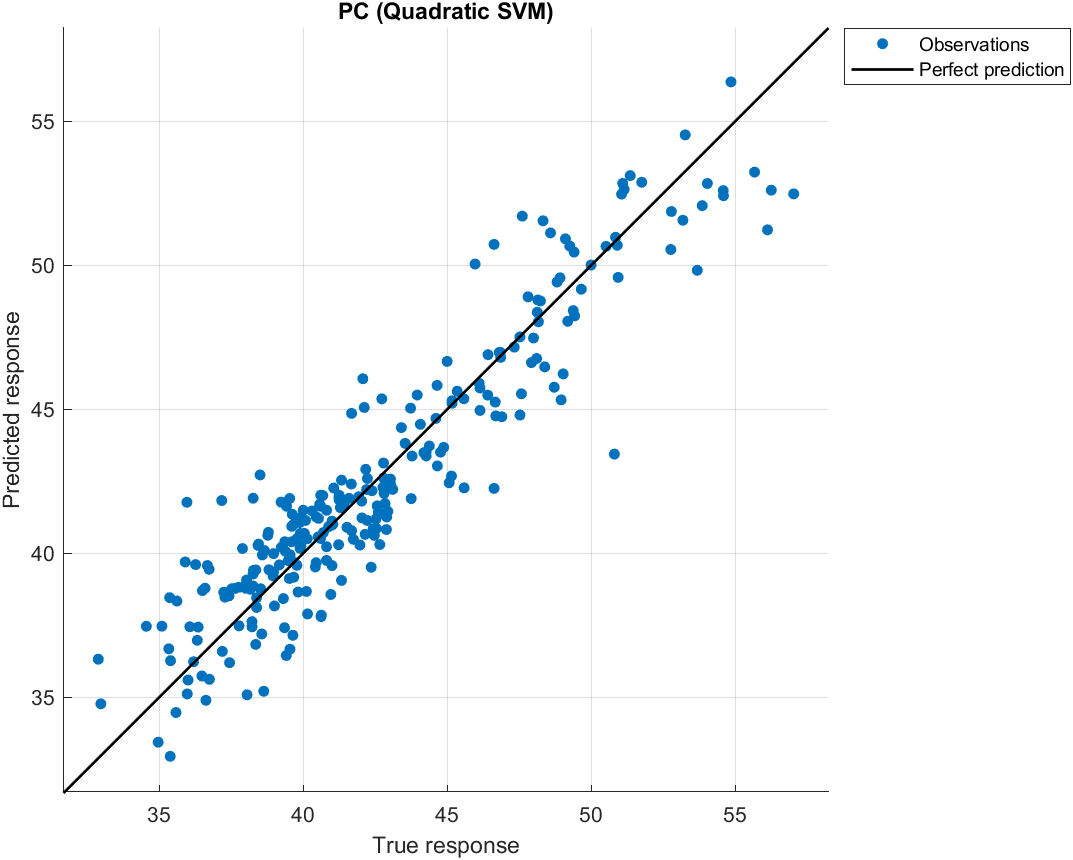

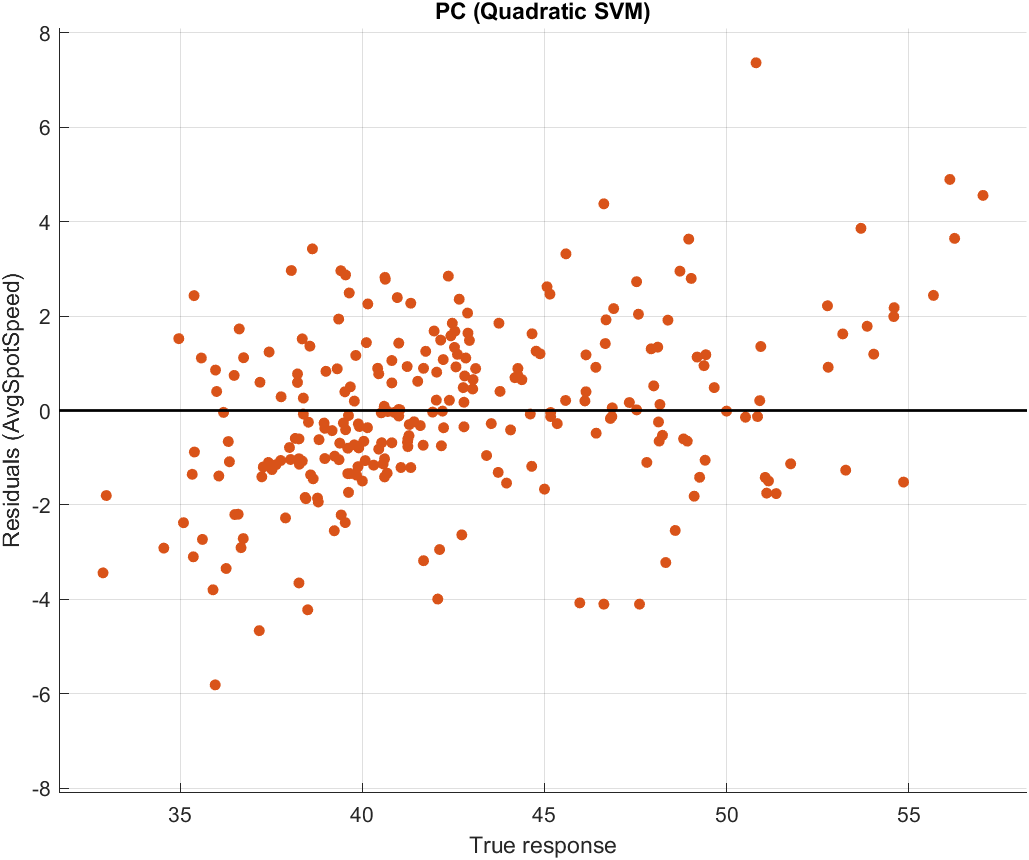


## Figure ‎A.10 PC SVR Summary


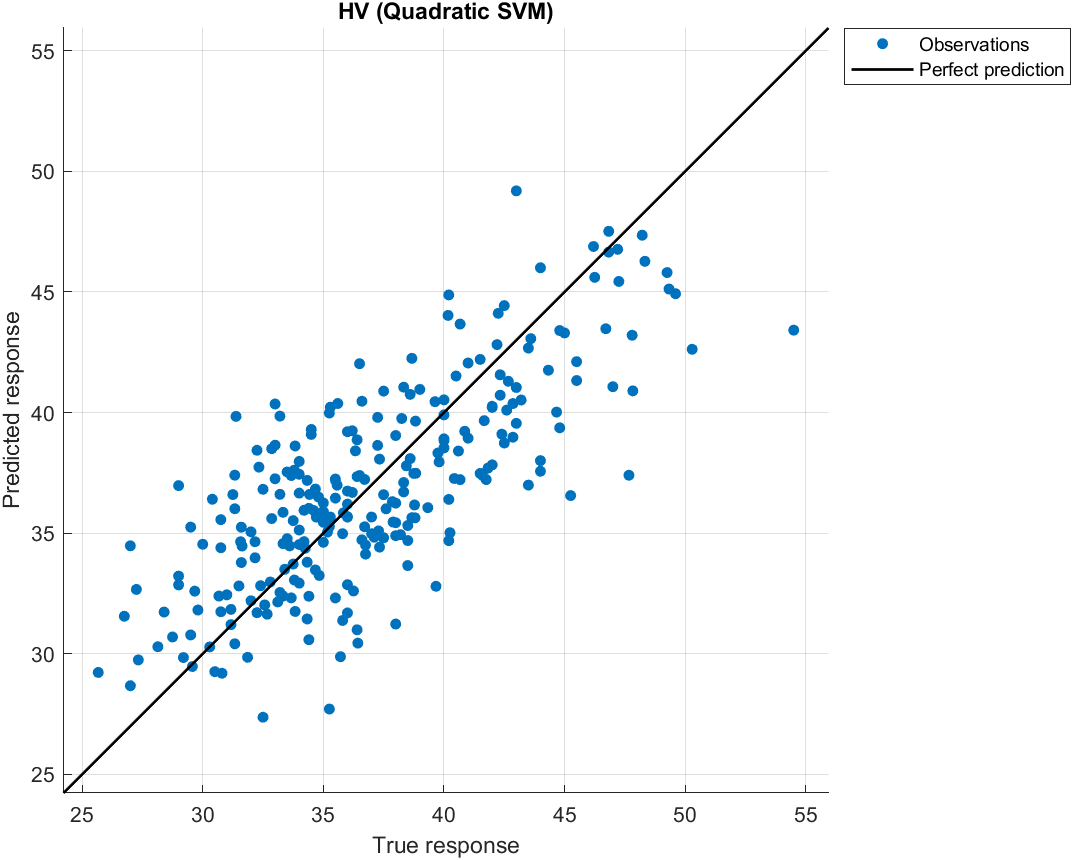

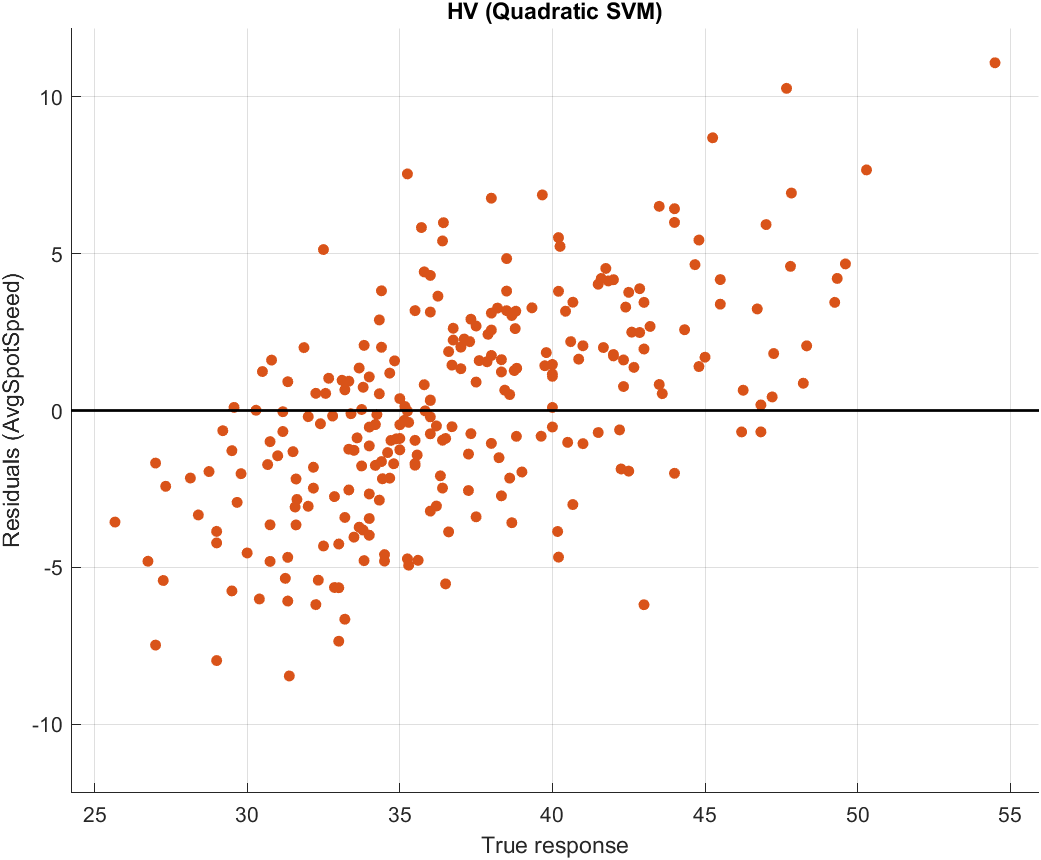

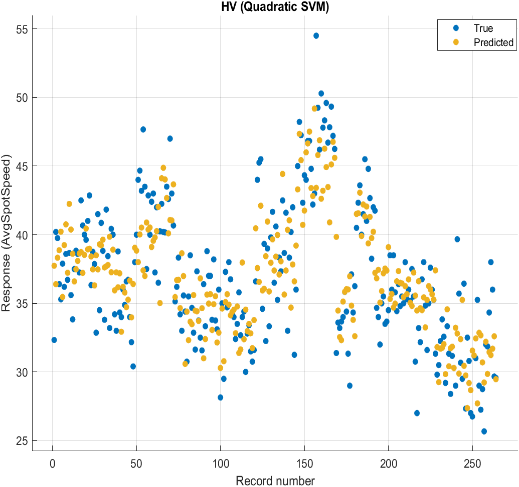


## Figure ‎A.11 HV SVR Summary


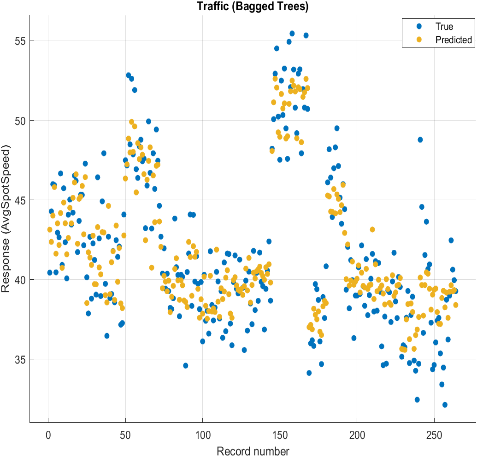

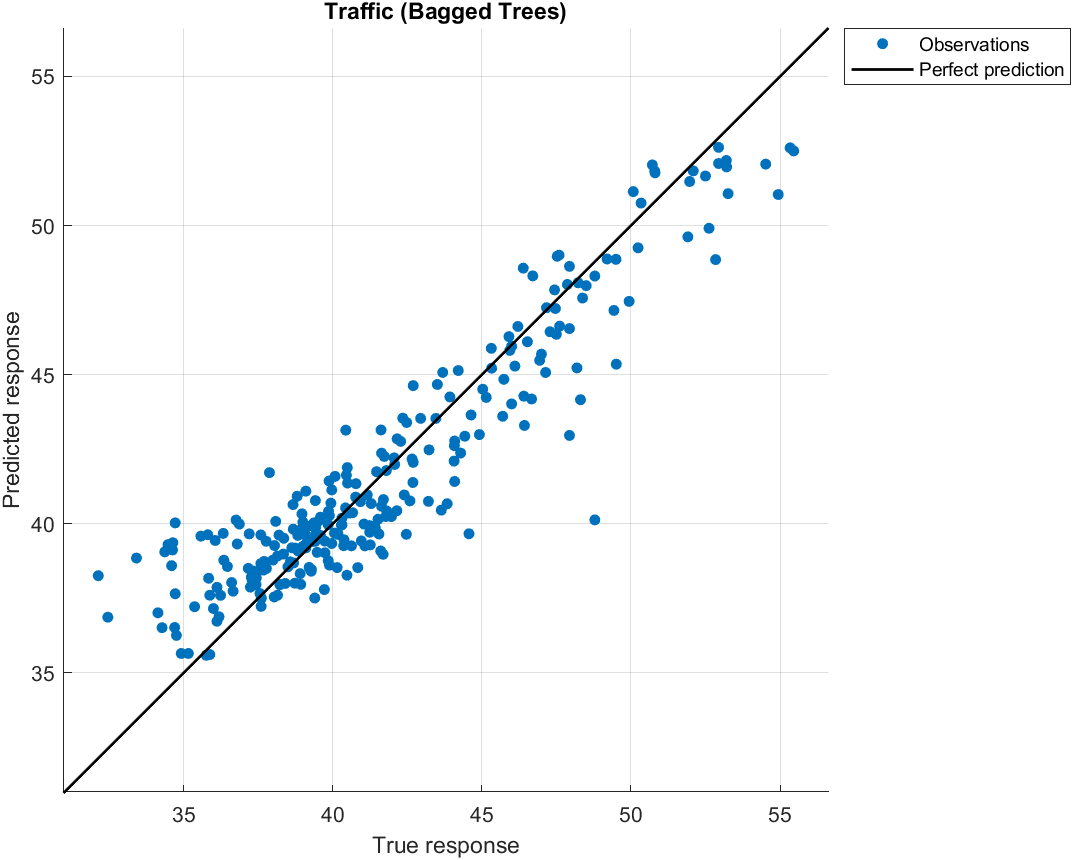

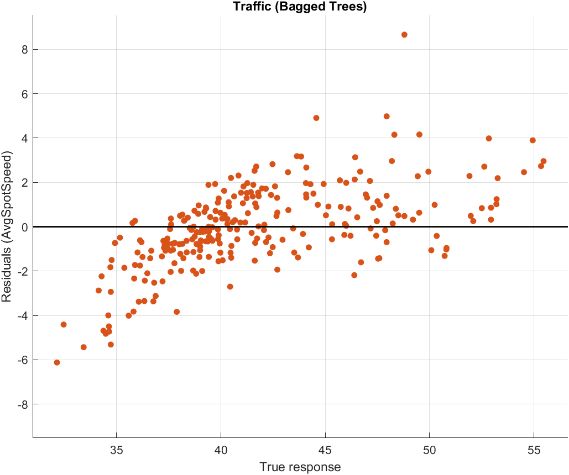


## Figure ‎A.12 Traffic RF Summary


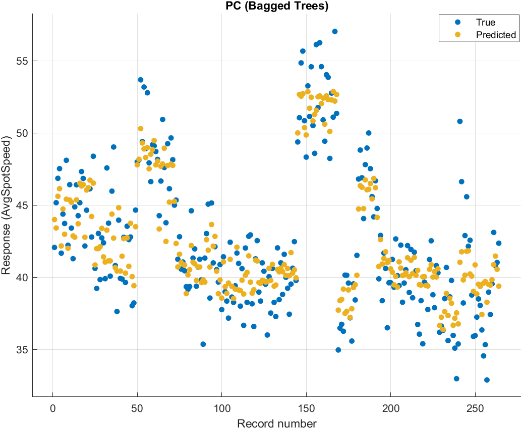

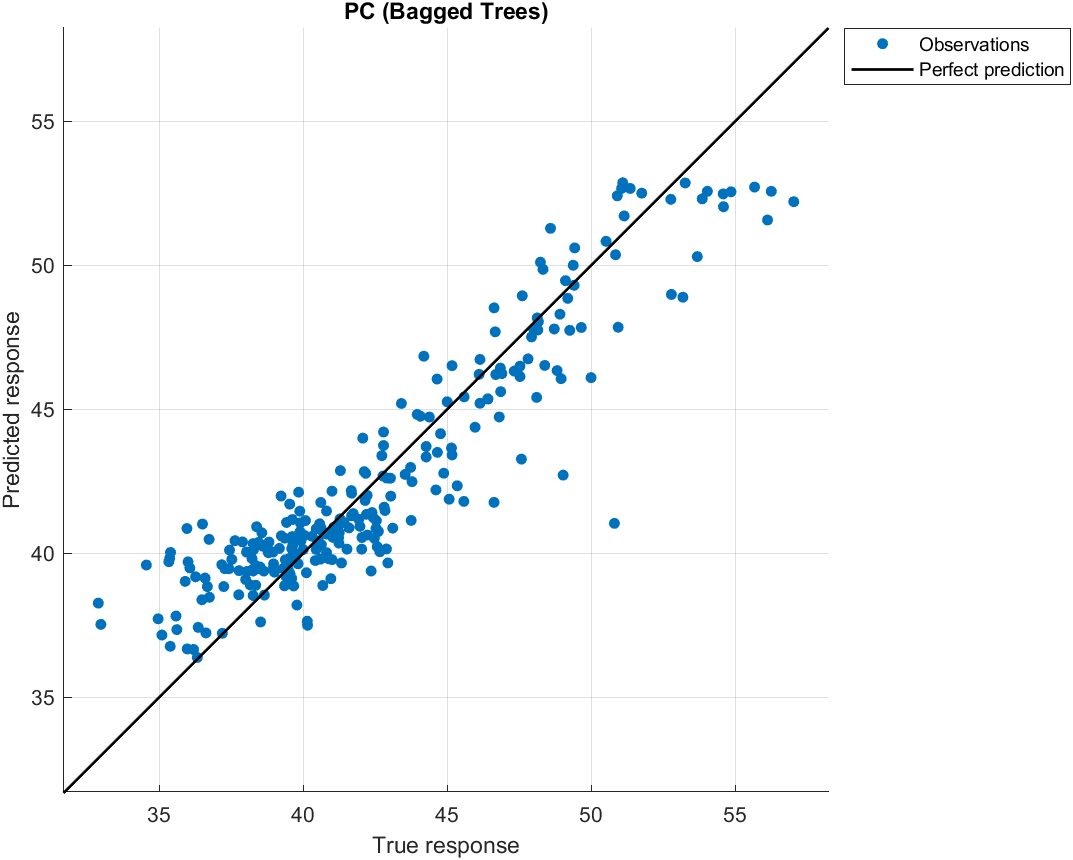

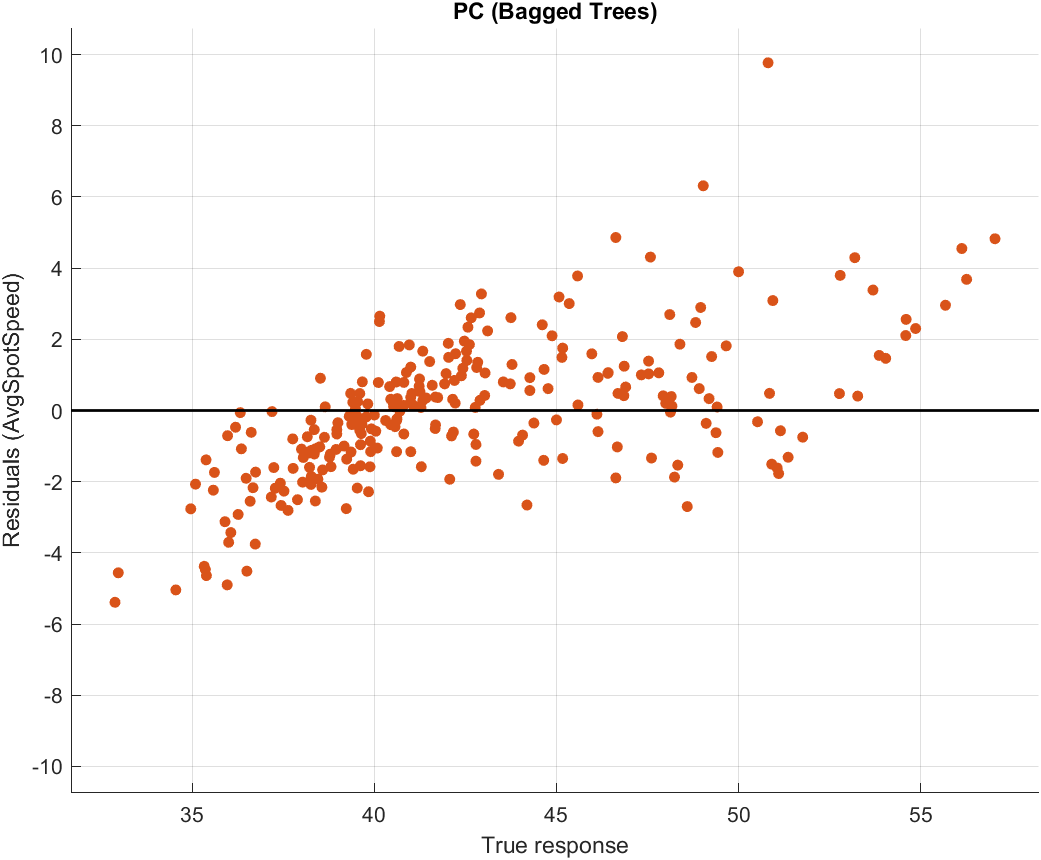


## Figure ‎A.13 PC RF Summary


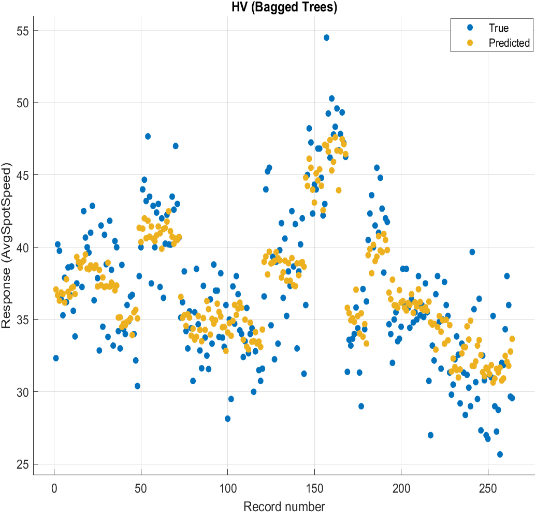

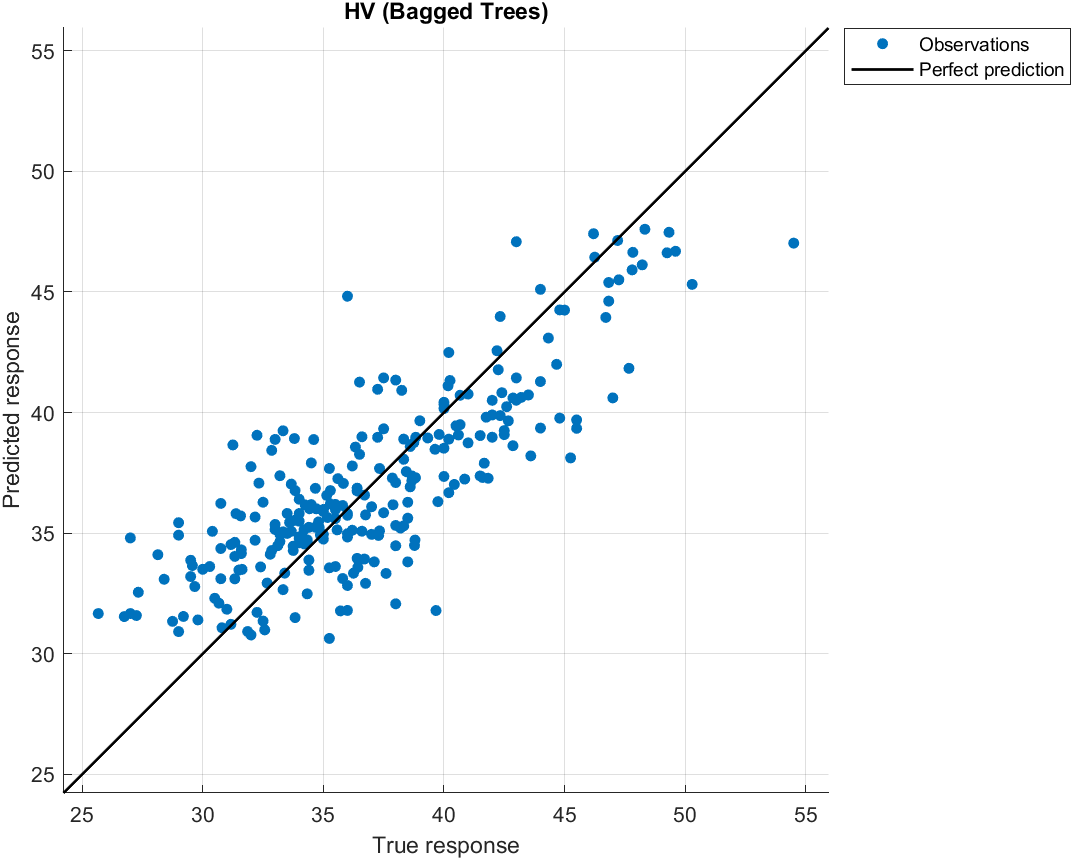

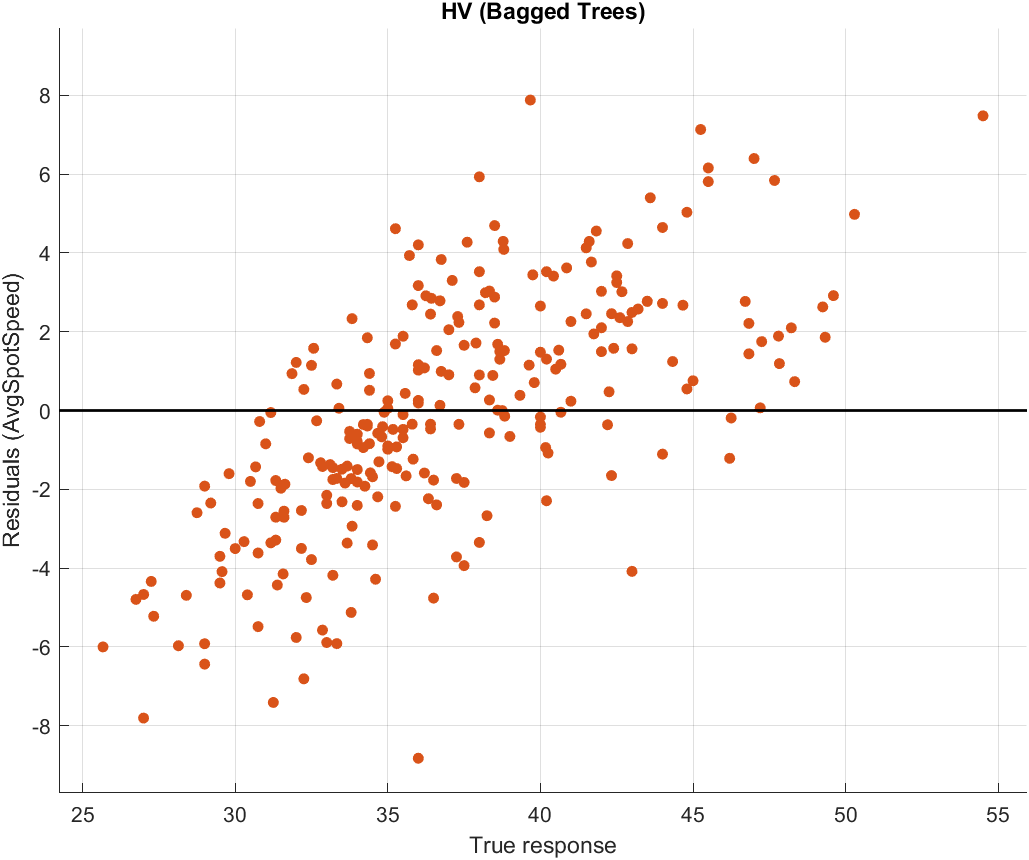


## Figure ‎A.14 HV RF Summary
